# Marginal zone B cells produce ‘natural’ atheroprotective IgM antibodies in a T cell–dependent manner

**DOI:** 10.1093/cvr/cvae027

**Published:** 2024-02-21

**Authors:** James Harrison, Stephen A Newland, Wei Jiang, Despoina Giakomidi, Xiaohui Zhao, Marc Clement, Leanne Masters, Andrej Corovic, Xian Zhang, Fabrizio Drago, Marcella Ma, Maria Ozsvar Kozma, Froher Yasin, Yuta Saady, Hema Kothari, Tian X Zhao, Guo-Ping Shi, Coleen A McNamara, Christoph J Binder, Andrew P Sage, Jason M Tarkin, Ziad Mallat, Meritxell Nus

**Affiliations:** Department of Medicine, University of Cambridge, Cambridge Biomedical Campus, Cambridge, UK; Department of Medicine, University of Cambridge, Cambridge Biomedical Campus, Cambridge, UK; Department of Medicine, University of Cambridge, Cambridge Biomedical Campus, Cambridge, UK; Department of Medicine, University of Cambridge, Cambridge Biomedical Campus, Cambridge, UK; Department of Medicine, University of Cambridge, Cambridge Biomedical Campus, Cambridge, UK; Department of Medicine, University of Cambridge, Cambridge Biomedical Campus, Cambridge, UK; Laboratory for Vascular Translational Sciences (LVTS), Université de Paris, INSERM U1148, Paris, France; Department of Medicine, University of Cambridge, Cambridge Biomedical Campus, Cambridge, UK; Department of Medicine, University of Cambridge, Cambridge Biomedical Campus, Cambridge, UK; Department of Medicine, Brigham and Woman’s Hospital, Harvard Medical School, Boston, MA, USA; Division of Cardiovascular Medicine, Department of Medicine, University of Virginia, Charlottesville, VA, USA; Wellcome-MRC Institute of Metabolic Science and Medical Research Council Metabolic Diseases Unit, University of Cambridge, UK; Department of Laboratory Medicine, Medical University of Vienna, Vienna, Austria; Department of Medicine, University of Cambridge, Cambridge Biomedical Campus, Cambridge, UK; Department of Medicine, University of Cambridge, Cambridge Biomedical Campus, Cambridge, UK; Division of Cardiovascular Medicine, Department of Medicine, University of Virginia, Charlottesville, VA, USA; Department of Medicine, University of Cambridge, Cambridge Biomedical Campus, Cambridge, UK; Department of Medicine, Brigham and Woman’s Hospital, Harvard Medical School, Boston, MA, USA; Division of Cardiovascular Medicine, Department of Medicine, University of Virginia, Charlottesville, VA, USA; Department of Laboratory Medicine, Medical University of Vienna, Vienna, Austria; Department of Medicine, University of Cambridge, Cambridge Biomedical Campus, Cambridge, UK; Department of Medicine, University of Cambridge, Cambridge Biomedical Campus, Cambridge, UK; Department of Medicine, University of Cambridge, Cambridge Biomedical Campus, Cambridge, UK; PARCC Inserm U970, Universite de Paris, Paris, France; Department of Medicine, University of Cambridge, Cambridge Biomedical Campus, Cambridge, UK

**Keywords:** B cells, T cells, Atherosclerosis, Antibodies, Interleukin-18

## Abstract

**Aims:**

The adaptive immune response plays an important role in atherosclerosis. In response to a high-fat/high-cholesterol (HF/HC) diet, marginal zone B (MZB) cells activate an atheroprotective programme by regulating the differentiation and accumulation of ‘poorly differentiated’ T follicular helper (Tfh) cells. On the other hand, Tfh cells activate the germinal centre response, which promotes atherosclerosis through the production of class-switched high-affinity antibodies. We therefore investigated the direct role of Tfh cells and the role of IL18 in Tfh differentiation in atherosclerosis.

**Methods and results:**

We generated atherosclerotic mouse models with selective genetic deletion of Tfh cells, MZB cells, or IL18 signalling in Tfh cells. Surprisingly, mice lacking Tfh cells had increased atherosclerosis. Lack of Tfh not only reduced class-switched IgG antibodies against oxidation-specific epitopes (OSEs) but also reduced atheroprotective natural IgM-type anti-phosphorylcholine (PC) antibodies, despite no alteration of natural B1 cells. Moreover, the absence of Tfh cells was associated with an accumulation of MZB cells with substantially reduced ability to secrete antibodies. In the same manner, MZB cell deficiency in *Ldlr^−/−^* mice was associated with a significant decrease in atheroprotective IgM antibodies, including natural anti-PC IgM antibodies. In humans, we found a positive correlation between circulating MZB-like cells and anti-OSE IgM antibodies. Finally, we identified an important role for IL18 signalling in HF/HC diet–induced Tfh.

**Conclusion:**

Our findings reveal a previously unsuspected role of MZB cells in regulating atheroprotective ‘natural’ IgM antibody production in a Tfh-dependent manner, which could have important pathophysiological and therapeutic implications.

## Graphical Abstract

**Graphical Abstract:**
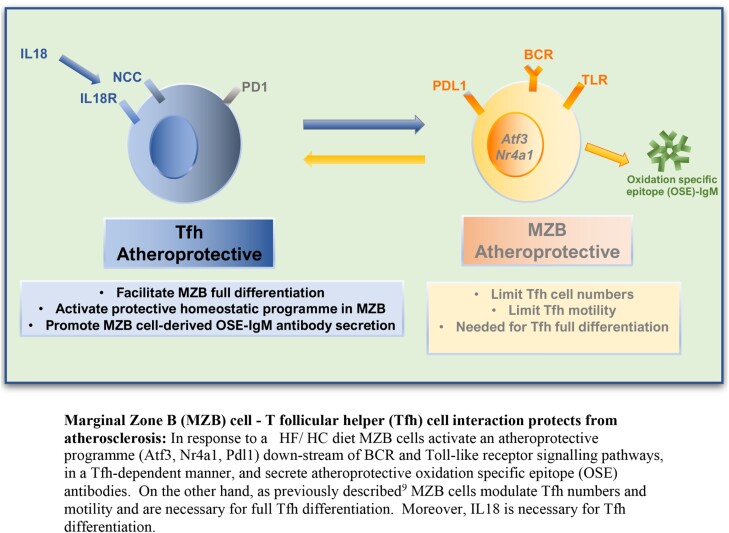

**Time of primary review: 19 days**

## 1. Introduction

Atherosclerosis is an arterial pathology with multiple genetic and environmental risk factors, initiated in response to trapping of low-density lipoproteins (LDLs) in the intima and their acquisition of inflammatory and immunogenic properties. The subsequent immune response involves interactions between vascular and circulating cells and mediators. Broad evidence supports the inflammatory theory of atherosclerosis, and innate and adaptive immune cells have been shown to participate in all stages of the disease.^1,2^

T follicular helper (Tfh) cells are specialized T cells that can be distinguished from other T helper populations for the expression of CXCR5, PD1 and ICOS surface markers, and their signature transcription factor, B cell lymphoma 6 (BCL6). They are the key orchestrators of the germinal centre (GC) reaction through their support of follicular B cell proliferation, somatic hypermutation, and class switch recombination, leading to the secretion of high-affinity antibodies and the formation of long-lived plasma and memory B cells.^3^ They are also involved in the extrafollicular plasma B cell response.^4^ Both the role of GC B cells^5,6^ and the role of Tfh cells^7–10^ remain controversial arguing for context-dependent properties of these cells in atherosclerosis. We have previously shown that *Ldlr^−/−^* mice with genetic deletion of marginal zone B (MZB) cells,^9^ which accumulate high numbers of ‘poorly differentiated’ Tfh cells resembling pre-Tfh,^9^ promote atherosclerosis. Using anti-ICOS-L antibodies to deplete Tfh in both *ApoE^−/−^* ^8^ and *Ldlr^−/−^* ^9^ mice did not impact atherosclerosis, but using the same strategy to reduce Tfh in mouse models with exacerbated Tfh/pre-Tfh cells was associated with decreased atherosclerosis. Thus, the role of the ‘normal’ Tfh response in atherosclerosis remains unclear.

Furthermore, previous RNA-seq from our ‘poorly differentiated’ Tfh vs. ‘normal’ Tfh cells identified *Il18r* as a potential driver of Tfh differentiation.^9^ IL18 is a pro-atherogenic cytokine^11^ that belongs to the IL1 family, and after the successful CANTOS trial results demonstrating the benefit of targeting IL1β in coronary artery disease (CAD), IL18 has been postulated as an alternative target.^12^ Its pro-atherogenic role in mice so far was attributed to its effect on Th cell differentiation and IFNγ production,^13^ but its role in Tfh differentiation has not been studied before.

## 2. Methods

### 2.1 Animals

All experiments were approved by the Home Office, UK (PPL PP9485757). The following strains of mice were used: *CD4^Cre/+^* ^14^ and *Rag2^−/−^* mice (Jackson Lab); *C57Bl6* and *Ldlr* ^−/−^ (Charles River); *Bcl6^flox/flox^* ^15^ (a gift to AS from Dr Harker); *Il18r^−/−^; NCC^−/−^* ^16^; *Cd79a^Cre/+^*; and *Rbpjk^flox/flox^*.^17,18^

For atherosclerosis studies, recipients were lethally irradiated and injected 10^7^ bone marrow (BM) cells i.v. After 4 weeks recovery,^19^ they were fed a high-fat/high-cholesterol (HF/HC; 21% fat, 0.15% cholesterol) diet for 8 or 16 weeks.

For IL18 studies, mice received i.p. IL18 (Biotechne) in PBS (2 μg/mouse/day) or PBS for 8 days. For immunization, 2 × 10^9^ sheep red blood cell (SRBC) was injected i.p.

At the end of the study, mice were euthanized by rising concentrations of CO_2_ inhalation in their cage (CO_2_ flow rate of 2 L/min for 5 min).

### 2.2 Flow cytometry

Single-cell suspensions of spleen, BM, para-aortic lymph node (PALN), and peritoneal lavage were stained with fluorophore-conjugated antibodies (see Supplementary material online, *Table S1*) and analysed using LSRII Fortessa (BD) flow cytometer. Dead cells were excluded based on FSc and SSc. Cell subsets were then identified as previously described^9,20^ (see Supplementary material online, *Table S2*). Examples of flow gating strategies are found in Supplementary material online, *Figure S1*.

### 2.3 Extent and composition of atherosclerotic lesions

The lesions in the root of the aorta beneath all three-valve leaflets were analysed with Masson’s trichrome or immunofluorescence as previously described.^9,20^ The antibodies to detect are macrophages (Mac-3, 1:200, Santa Cruz) and T cells (CD3, 1:100, Dako). Whole aortas were used in an *en face* preparation for oil red O staining. All was quantified with ImageJ as previously described.^9,20^

### 2.4 Determination of circulating antibodies

Specific antibody titres in plasma were determined by chemiluminescent ELISA as previously described.^21,22^

### 2.5 Determination of serum lipid levels

Total cholesterol, HDL-C, and triglycerides were measured using an enzymatic method in a Siemens Dimension RxL analyser.

### 2.6 Purification of MZB

For splenic MZB cell purification, first B cell–enriched populations were separated by EasySep B cell purification kit (StemCell). B cells were stained with anti-CD23-PE, anti-CD21-FITC, and 7-AAD for cell viability. MZBs (CD21^hi^CD23^low^) were sorted using Aria III Cell Sorter (BD). Purity was >95%.

### 2.7 *Ex vivo* MZB cell IgM production stimulation assay

A total of 250 000 sorted MZB cells/well were cultured for 3 days with CpG ODN (2 μM tlrl-1826, InvivoGen). IgM antibody levels in supernatants were measured using ELISA.

### 2.8 RNA sequencing

Splenic MZB cells were directly sorted in RLT Plus Micro RNA buffer for RNA extraction (Qiagen). RNA integrity number (RIN) values for all samples were >7. RNA (2.5 ng) was whole transcriptome amplified using Ovation RNA-seq System V2 (NuGEN). Two micrograms per sample of the amplified cDNA was used to generate sequencing library using Ovation Rapid DR Library System (NuGEN). Sequencing was performed on an Illumina HiSeq 2500 (CRUK, Cambridge). Bioinformatic analysis was performed as explained in Supplementary material online, *Methods*.

### 2.9 Quantitative RT-PCR

RNA from sorted MZB cells and splenic cells from fresh frozen optimal cutting temperature (OCT) compound embedded spleen sections were isolated using RNAeasy Plus micro kit (Qiagen). RT-PCR was performed using QuantiTect Reverse Transcription Kit (Qiagen) or SMART-Seq v4 (Takara Bio). Primer sequences in Supplementary material online, *Table S3*.

### 2.10 Human samples

Blood samples were analysed from participants with atherosclerosis enrolled in the following studies: (i) Residual Inflammation and Plaque Progression Long-Term Evaluation (RIPPLE, NCT04073810; *n* = 16); (ii) Rituximab in Patients With Acute ST-elevation MI Study (RITA-MI, NCT03072199^23^; *n* = 21); and (iii) Coronary Assessment in Virginia cohort (CAVA, *n* = 20; see Supplementary material online, *Table S4*). Participants from RIPPLE (2 weeks) and RITA-MI (48 h) had recent myocardial infarction (MI). Participants in the CAVA study had stable CAD. Participants gave written consent in accordance with the protocol approved by the local research ethics committee in the UK (19/EE/0043; 16/EE/0241) or the institutional review board at the University of Virginia, USA (IRB HSR #15328), in accordance with the Declaration of Helsinki and UK Human Tissue Act 2004.

### 2.11 Statistical analysis

Values are expressed as means ± SEM. Where data sets passed normality tests, differences between values were examined using Student’s *t*-test or two-way analysis of variance (ANOVA). For experiments with four or less replicates, non-parametric Mann–Whitney test was used. For correlations, Spearman’s non-parametric test was applied. For EnrichR top canonical pathways and genes in the RNA-seq, Fisher’s exact test was used. In all figures, *P < 0.05, **P < 0.01, and ***P < 0.001.

## 3. Results

### 3.1 Absence of Tfh increases early atherosclerosis

To study the role of Tfh in atherosclerosis, we first followed a BM reconstitution methodology in *Ldlr^−/−^* mice, described in a previously published report on the role of Tfh cells in atherosclerosis.^7^ We generated mice with T cell–specific conditional deletion (*CD4^Cre/+^*) of the Tfh lineage transcription factor *Bcl6* (*Bcl6^flox/flox^).* We then reconstituted lethally irradiated *Ldlr^−/−^* mice with a BM containing 100% cells from *CD4^Cre/+^; Bcl6^flox/flox^* (that are unable to generate Tfh). Our control mice received a BM containing 80% cells from *CD4^Cre/+^; Bcl6^flox/flox^* + 20% cells from *CD4^+/+^; Bcl6^flox/flox^* (which reconstitute *CD4^+/+^; Bcl6^flox/flox^* WT Tfh). After recovery, mice were put on a HF/HC western diet for 8 weeks (see Supplementary material online, *Figure S2A*). This time point was chosen based on the fact that in mouse models of HF/HC-induced atherosclerosis, adaptive immunity plays a major role in the early phase of the disease but becomes less important in the late phase with extended duration of HF/HC diet and severe hypercholesterolaemia.^24^  *Ldlr^−/−^* mice transplanted with *CD4^Cre/+^; Bcl6^flox/flox^* mice BM showed only a partial (50%) reduction in Tfh (see Supplementary material online, *Figure S2B*), suggesting that, despite ‘lethal’ irradiation, the recipient host (which is competent for Tfh generation) was able to reconstitute a large number of Tfh. The presence of recipient T cell progenitor cells resistant to irradiation has been previously shown.^25^ Surprisingly, despite a partial depletion of Tfh cells, these mice showed an unexpected significant increase of atherosclerotic plaque development in the aortic roots (see Supplementary material online, *Figure S2C*), suggesting that Tfh may have a protective, not detrimental, role in atherosclerosis.

To track the specific Tfh reconstitution in lethally irradiated mice, we decided to use the CD45 (cluster of differentiation 45) congenic lineage tracing system. Lethally irradiated *CD45.2^+^ CD45.1^−^ CD4^Cre/+^; Bcl6^flox/flox^* (No Tfh) and *CD45.2^+^ CD45.1^−^ CD4^+/+^; Bcl6^flox/flox^* (WT) recipient mice were reconstituted with 80% *CD45.2^+^ CD45.1^−^ CD4^Cre/+^; Bcl6^flox/flox^* + 20% *CD45.2^−^ CD45.1^+^* BM. After recovery, mice were injected with SRBCs to induce the activation of the Tfh-GC response^26^ (see Supplementary material online, *Figure S2D*). Specific Tfh reconstitution from the BM (80% of CD4^+^ CD44^+^ CXCR5^+^ PD1^+^ Tfh coming from the *CD45.2^−^ CD45.1^+^* donor) was successful only when the recipient was deficient for Tfh (*CD45.2^+^ CD45.1^−^ CD4^Cre/+^; Bcl6^flox/flox^*). The BM was unable to successfully reconstitute Tfh in WT recipients (CD45.2^+^ CD45.1^−^  *CD4^+/+^; Bcl6^flox/flox^*). Most of the reconstituted Tfh in the latter mice were still from the recipient host (see Supplementary material online, *Figure S2E*). Our data are in agreement with the published literature that T cells are unlikely to reconstitute fully from the donor BM if the recipient is T cell competent.^25^

**Figure 1:**
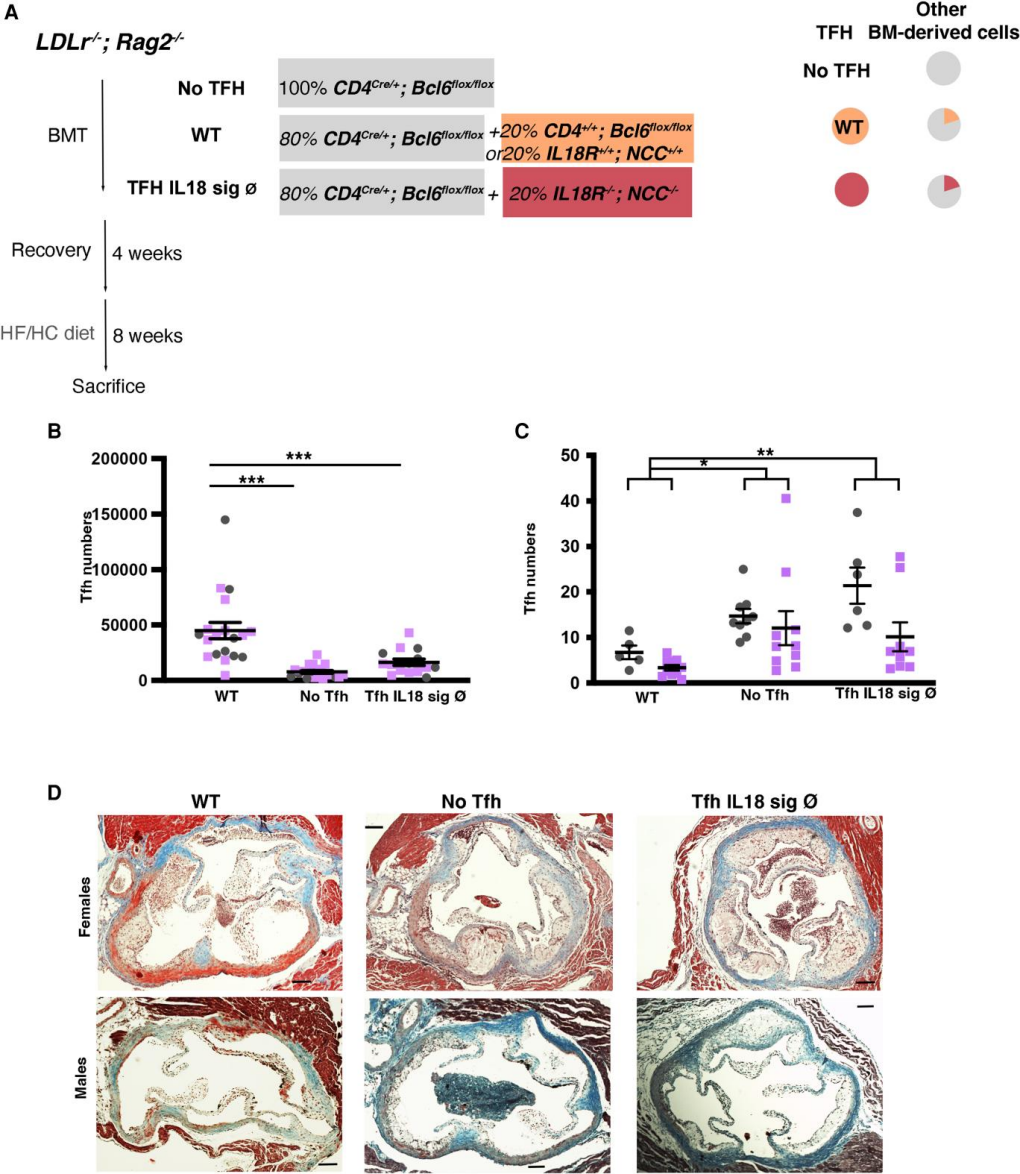
Tfh cell deficiency and the absence of IL18 signalling in Tfh cells accelerate the development of atherosclerosis in mice. Data from males and females Ldlr^−/−^; Rag2^−/−^ after BM transplant with 100% CD4^Cre/+^; Bcl6^flox/flox^ (No Tfh); 80% CD4^Cre/+^; Bcl6^flox/flox^ + 20% CD4^+/+^; Bcl6^flox/flox^; or 20% Il18r^+/+^; NCC^+/+^ (WT) and 80% CD4^Cre/+^; Bcl6^flox/flox^ + 20% Il18r^−/−^; NCC^−/−^ (Tfh Il18 sign Ø) fed a HF/HC diet for 8 weeks. Represented data from five experiments. WT group includes mice that are either CD4^+/+^; Bcl6^flox/flox^ (littermates to the No TFH group) or Il18r^+/+^; NCC^+/+^ WT (littermates to Il18r^−/−^; NCC^−/−^ mice) and were combined to reduce the number of animals used in these experiments (3Rs). (*A*) Schematic diagram of the experiment. (*B*) Total splenic Tfh cells (CD4^+^ CD44^hi^ CXCR5^+^ PD1^+^; *n* = 6–10 mice/group). (*C*) Quantification of atherosclerotic plaque area in aortic roots. (*D*) Representative images of Masson’s trichrome staining (original magnification ×10; scale bars: 200 μm). Each symbol represents an individual mouse; horizontal bars denote mean ± SEM. (*B*) Student’s *t*-test and (*C*) two-way ANOVA. *P < 0.05, **P < 0.01, and ***P < 0.001.

To obtain an atherosclerotic mouse model with a specific deletion of Tfh, we reconstituted lethally irradiated *Ldlr^−/−^; Rag2^−/−^ mice* (no T cells and no B cells, to avoid reconstitution with irradiation-resistant recipient T cells) with a BM containing 100% *CD4^Cre/+^; Bcl6^flox/flox^* (No Tfh group) or a mixed BM chimaera containing 80% *CD4^Cre/+^; Bcl6^flox/flox^* + 20% *CD4^+/+^; Bcl6^flox/flox^* (WT Tfh group; *Figure 1A*). Additional lethally irradiated *Ldlr^−/−^; Rag2^−/−^* mice were reconstituted with 80% *CD4^Cre/+^; Bcl6^flox/flox^* + 20% *Il18r^−/−^; NCC^−/−^* (Tfh Il18 sign Ø) or 20% *Il18r^+/+^; NCC^+/+^* (WT group) to study the role of the IL18 signalling pathway in Tfh (see the last chapter of Results). After recovery, mice were put on a HF/HC diet for 8 weeks. There was almost a full depletion of splenic (*Figure 1B*; Supplementary material online, *Figure S3A*) and PALN (see Supplementary material online, *Figure S3B*) Tfh cells in the No Tfh group. All the other T cell subsets, including splenic T effector memory (TEM), T regulatory (Treg), and T follicular regulatory (Tfr), and both splenic and PALN Th17 and Th1 cells were not significantly altered in WT vs. No Tfh mice (see Supplementary material online, *Figure S3C–I*). Numbers of splenic neutrophils, macrophages, Ly6C^hi^ and Ly6C^lo^ monocytes, and eosinophils were not different between both groups (see Supplementary material online, *Figure S4A–E*).

Lack of Tfh led to a striking acceleration of atherosclerosis in aortic roots (*Figure 1C* and *D*) and aortic arches (see Supplementary material online, *Figure S5A*), confirming an unsuspected atheroprotective role for Tfh. The substantial increase in atherosclerosis could not be explained by changes in serum lipid levels (see Supplementary material online, *Figure S5B–D*). Plaque macrophage content was not significantly different between WT and No Tfh mice (see Supplementary material online, *Figure S6A–C*). In No Tfh mice, there was a significant increase of CD3^+^ T cells in both intima and adventitia compared with WT mice (see Supplementary material online, *Figure S6D–F*). Plaque collagen content and necrotic core size were significantly increased in the No Tfh group compared to the WT group (see Supplementary material online, *Figure S6G* and *H*).

The difference in plaque size between both groups was smaller after extended duration (16 weeks) of HF/HC diet (see Supplementary material online, *Figure S7A–D*), consistent with the predominant role of adaptive immunity in the early stages of atherosclerosis in these murine models.^24^

### 3.2 Tfh are necessary for the secretion of IgM natural antibodies

**Figure 2:**
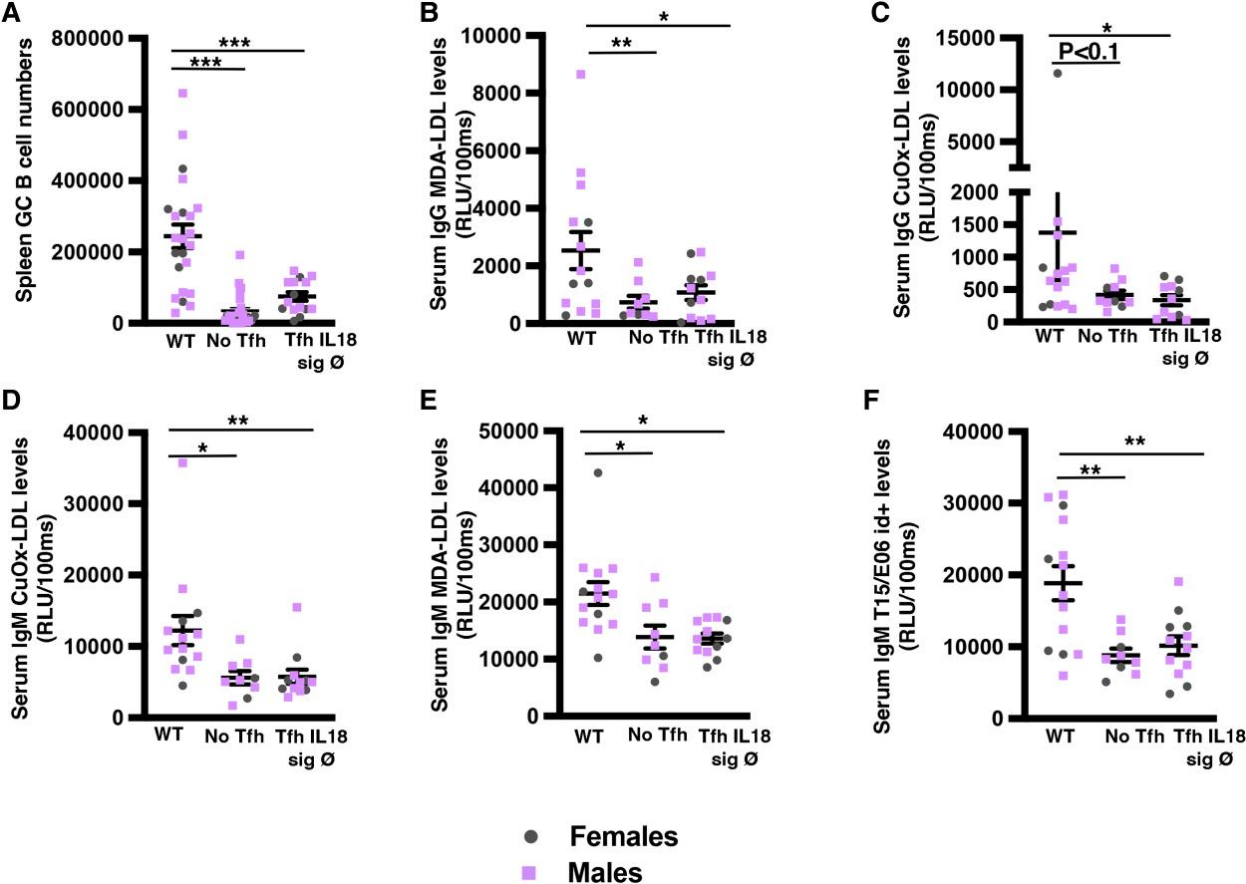
Lack of Tfh cells and absence of IL18 signalling in Tfh lead to a profound reduction of anti-OSE IgG and IgM antibodies, including ‘natural’ IgM antibodies. Data from the same experimental groups as in *Figure 1*. (*A*) Total numbers of splenic GC B cells (B220^+^ Gl7^hi^ CD95^hi^; *n* = 6–11 mice/group). (*B–F*) Graphs showing total serum subtypes of IgG (*B*, *C*) and IgM (*D–F*) levels. Student’s *t*-test. *P < 0.05, **P < 0.01, and ***P < 0.001.

As expected, splenic and PALN GC B cells were profoundly reduced in No Tfh compared with WT Tfh mice (*Figure 2A*; see Supplementary material online, *Figure S8A* and *B*), which was associated with a reduction of plasma cells in the BM (see Supplementary material online, *Figure S8C*). Concomitantly, there was a significant reduction of serum IgG antibodies against malondialdehyde (MDA)-LDL and almost significant for IgG anti–copper-oxidized (CuOx)-LDL (*Figure 2B* and *C*). MDA and CuOx-LDL IgM antibodies were also significantly reduced in No Tfh vs. WT Tfh mice (*Figure 2D* and *E*). Surprisingly, mice with No Tfh also had significantly lower levels of the previously defined B1 cell–derived natural IgM T15/E06 idiotype+ (id+) antibody directed against the oxidation-specific epitope (OSE) phosphorylcholine (PC; *Figure 2F*) while levels of B1a and B1b peritoneal cells were similar between the two groups (see Supplementary material online, *Figure S8D*). Other reported positive regulators of IgM levels in atherosclerosis like IL5 and BAFF were significantly upregulated in spleens of No Tfh compared to WT Tfh mice (see Supplementary material online, *Figure S8E–H*). These data suggest that at least part of the ‘natural’ IgM anti-PC antibody production is dependent on Tfh activation and may explain the acceleration of early atherosclerosis in the absence of Tfh.

### 3.3 The absence of Tfh leads to ‘aberrant’ MZB cells

**Figure 3:**
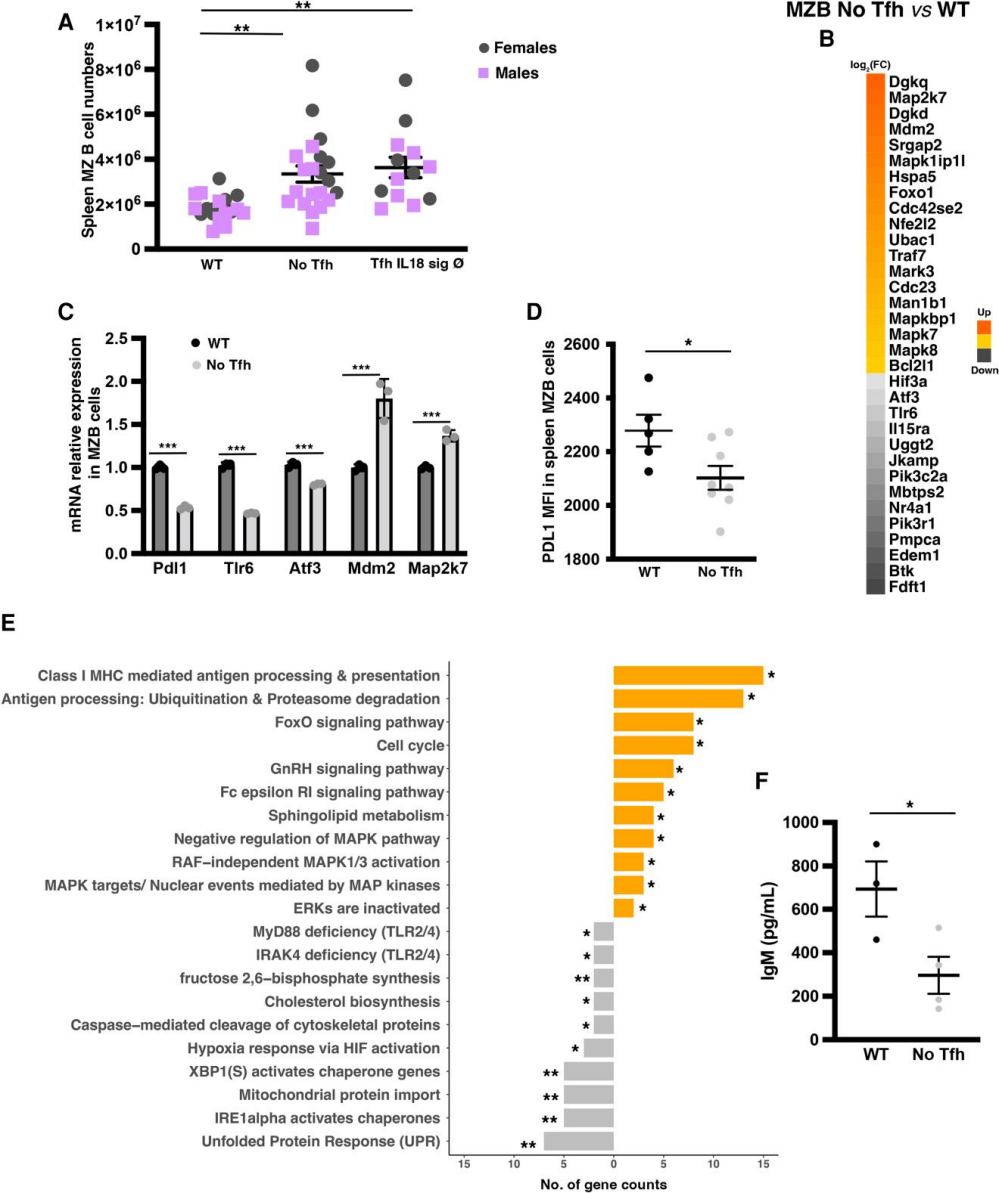
Lack of Tfh cells leads to accumulation of ‘aberrant’ MZB cells. Data from the same experimental No Tfh and WT groups. (*A*) Total splenic MZB cells (*n* = 12–15 mice/group). (*B*, *C*, and *E*) MZB cell RNA-seq from No Tfh and WT groups (*n* = 5–6 mice/group). (*B*) Clustered heat map of 33 genes that were differentially expressed. (*C*) qRT-PCR for Pdl1 (*n* = 3 mice/group). (*D*) PDL1 surface expression by flow cytometry. (*E*) Selected significantly enriched GSEA pathways. Each bar represents the number of significantly expressed genes in each pathway. Orange denotes up- and grey downregulated in MZB cells from No Tfh vs. WT. (*F*) IgM levels in the supernatants of sorted MZB cells (*n* = 6–9 mice/group) after culture with CpG. Representative plot from two independent experiments with similar results. *P* < 0.05 (Student’s *t*-test) and *P* < 0.1 (Mann–Whitney). (*A*, *C*, *D*, and *F*) Student’s *t*-test and (*E*) Fisher’s exact test. *P < 0.05, **P < 0.01, and ***P < 0.001.

Given our previous work demonstrating direct interaction between (pre-)Tfh and MZB cells,^9^ which are innate-like B cells and the main producers of IgM antibodies,^27^ we examined changes in MZB cells. Splenic total and follicular B cell numbers were similar between the two groups (see Supplementary material online, *Figure S8H* and *I*), but lack of Tfh was associated with a significant increase in MZB cell numbers (*Figure 3A*). Intrigued by this finding and to gain additional mechanistic insight, we performed *RNA-seq* on sorted splenic MZB cells after 8 weeks of HF/HC diet in WT vs. No Tfh groups. We first focused on a subset of genes that we have shown previously to be required for the activation of an atheroprotective programme in MZB cells in response to HF/HC diet.^9,20^ MZB cells from No Tfh mice had a significant decrease of transcription factors *Atf3* and *Nr4a1*, and the surface protein PDL1, as well as their upstream regulators, including the B cell receptor and Toll-like receptor (*Btk*, *Tlr6*) signalling pathways (*Figure 3B–D*).

Further analysis of the *RNA-seq* data revealed a downregulation in the expression of the master regulators necessary for the formation and activation of antibody-secreting plasma B cells^28^ (*Figure 3E*). There was a significant decrease in the unfolded protein response (e.g. *Edem1*, *Atf3*, and *Ppp2r5b*), endoplasmic-inducing stress (e.g. IRE1 alpha activated chaperones: *Ddx11* and *Extl3*), and *XBP1* signalling pathways (e.g. *Srpr*, *Edem1*, and *Add1*), providing a plausible explanation for the significant decrease of IgM antibody production in mice with No Tfh. On the other hand, MZB cells from No Tfh showed significant upregulation of genes related to proliferation (e.g. *Mapk8* and *Mapk7*) and cell cycle (e.g. *Cdc23*, *Cdkn2*, and *E2f2*) pathways, which might be activated independently of BCR signalling pathway, causing the accumulation of these ‘aberrant’ MZB cells. Thus, lack of Tfh leads to the accumulation of ‘aberrant’ MZB cells that are unable to activate their atheroprotective programme and form antibody-secreting cells.

To test this hypothesis, sorted splenic MZB cells from WT Tfh vs. No Tfh groups after 8 weeks on HF/HC diet were cultured with CpG (to stimulate IgM production). MZB cells from No Tfh mice secrete significantly less IgM antibodies than those from WT mice (*Figure 3F*), and this was associated with decrease PDL1 expression (see Supplementary material online, *Figure S9A* and *B*) confirming the *in vivo* phenotype. Furthermore, MZB cells from SRBC immunized *Cd4^Cre/+^*; *Bcl6^flox/flox^* (No Tfh) mice also showed significant reduced IgM secretion compared to MZB cells from *Cd4^+/+^*; *Bcl6^flox/flox^* (WT) mice (see Supplementary material online, *Figure S9C*), supporting a Tfh-dependent MZB cell antibody secretion in different clinical contexts.

### 3.4 MZB cells produce atheroprotective IgM antibodies

**Figure 4:**
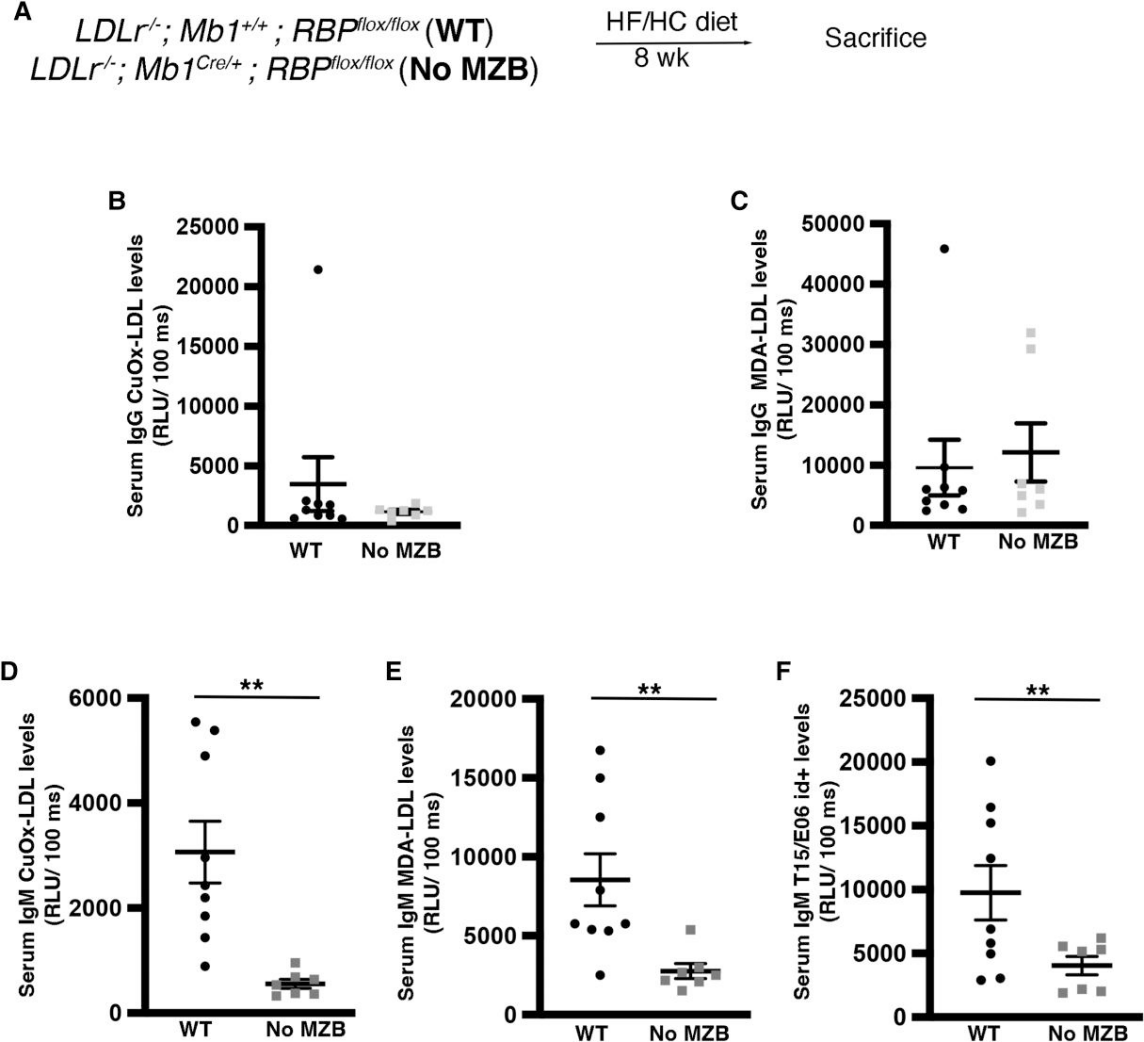
MZB cells are necessary for the formation of IgM natural antibodies. Males and females Ldlr^−/−^; Cd79a^Cre/+^; RBP^flox/flox^ and Ldlr^−/−^; Cd79a^+/+^; RBP^flox/flox^ (WT) were fed a HF/HC diet for 8 weeks (*A–F*). (*A*) Schematic diagram of the experimental procedure. (*B–F*) Graphs showing total serum subtypes of IgG (*B–D*) and IgM (*E–G*) levels. Student’s *t*-test. **P < 0.01.

To address the role of MZB cells in the production of IgM antibodies in atherosclerosis, we created a new atherosclerotic mouse model with No MZB cells from birth: *Ldlr^−/−^; Cd79a^Cre/+^; RBP^flox/flox^* (No MZB) and *Ldlr^−/−^; Cd79a^+/+^; RBP^flox/flox^* (WT; *Figure 4A*). As previously shown,^9,29^ Notch signalling disruption selectively in B cells led to mice with No MZB (see Supplementary material online, *Figure S10A*), which, as expected,^9^ significantly increased Tfh cells after 8 weeks of a HF/HC diet (see Supplementary material online, *Figure S10B*). Tfh from *Ldlr^−/−^* mice with No MZB showed reduced CXCR5 and PD1 expression compared with WT mice (see Supplementary material online, *Figure S10C* and *D*), corroborating our previous finding that lack of MZB cells leads to the accumulation of ‘poorly differentiated’ Tfh.^9^ There was a tendency to increased GC levels (see Supplementary material online, *Figure S10E*) but IgG-CuOx-LDL and IgG-MDA-LDL were not different between the two groups (*Figure 4B* and *C*). However, absence of MZB cells led to a dramatic decrease of IgM anti-CuOx-LDL, IgM anti-MDA-LDL, and IgM T15/E06 id+ titres (*Figure 4D* and *F*). Thus, MZB cells substantially contribute to the production of atheroprotective ‘natural’ anti-OSE IgM antibodies.

### 3.5 Circulating human MZ-like B cells positively correlate with anti-OSE IgM antibody levels

**Figure 5:**
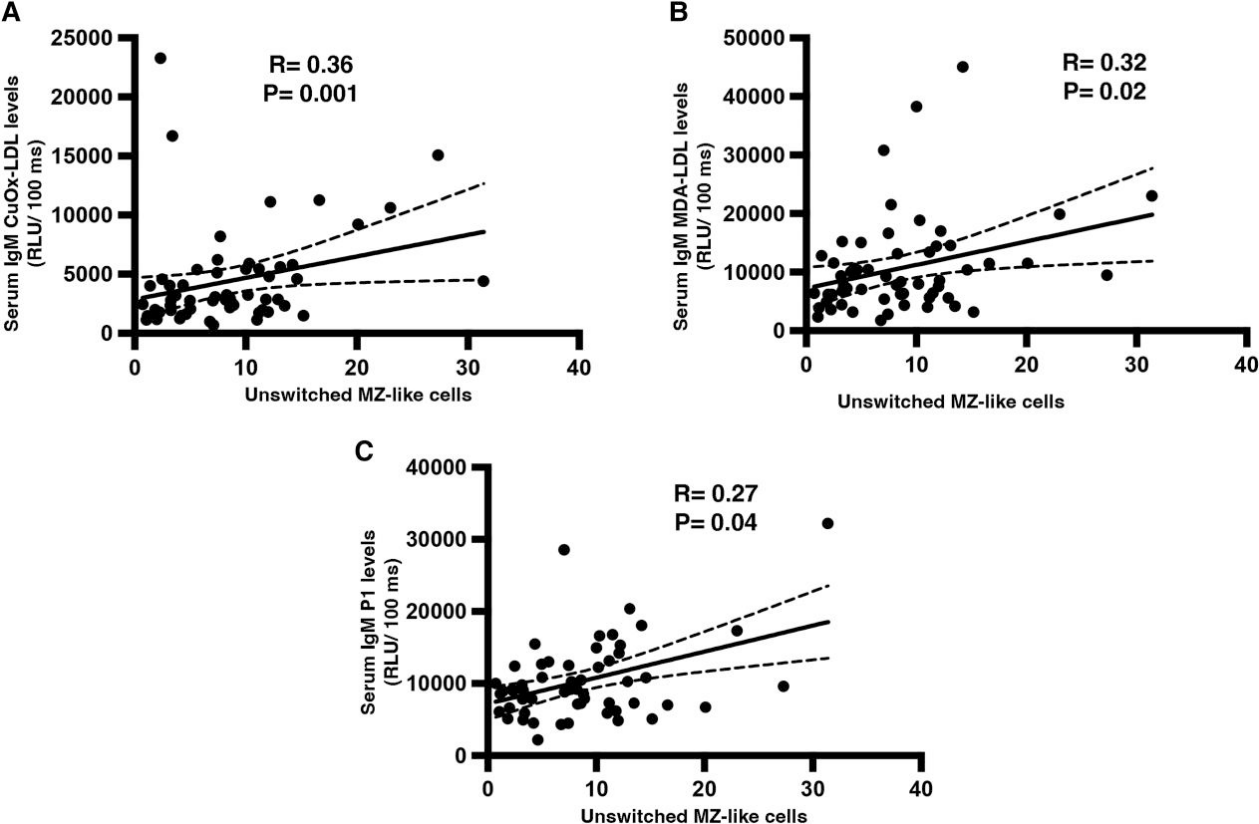
Circulating human MZ-like B cells positively correlate with anti-OSE IgM levels. PBMCs and serum were collected from patients of the RIPPLE (2 weeks post-MI), RITA-MI (2 days post-MI), and CAVA (stable CAD) populations (*n* = 57; *A–C*). Correlations between unswitched MZ-like B cells and (*A*) IgM CuOx-LDL, (*B*) IgM MDA-LDL, and (*C*) IgM-P1 antibodies. Spearman’s rank order correlation.

To explore the association between MZB cells and the levels of atheroprotective anti-OSE IgM antibodies in humans, we measured blood MZB-like cells and IgM antibodies in 57 patients with established CAD with or without a recent MI (see Supplementary material online, *Table S4*). Circulating MZB-like cells were defined as unswitched CD27^+^ IgD^+^ memory B cells^9,30^ (see Supplementary material online, *Table S2* and *Figure S1*). We found a significant positive correlation between circulating MZB-like cells and IgM antibodies against CuOx-LDL, MDA-LDL, and the P1 peptide mimotope of MDA-LDL (*Figure 5A–C*). MZ-like cells did not correlate with IgG levels for any of the OSE specificities checked (data not shown). This result further suggests a potential role of MZB cells as producers of anti-OSE IgM antibodies in humans.

### 3.6 The absence of IL18 signalling pathway in Tfh decreases their numbers and increases atherosclerosis

Re-analysis of our previous RNA-seq data on ‘poorly differentiated’ Tfh in the absence of MZB cells identified *Il18r* as one of the genes that were differentially expressed in these Tfh cells^9^ (see Supplementary material online, *Figure S11A*). Interestingly, both Tfh and IL18 serum levels were significantly increased in *Ldlr^−/−^* mice fed a HF/HCD compared with mice fed a control chow diet (see Supplementary material online, *Figure S11B* and *C*). Administration of IL18 to C57Bl/6 mice significantly increased Tfh cells, including in a widely validated model of SRBC-induced Tfh-GC formation (see Supplementary material online, *Figure S11D*). Therefore, we decided to address the role of IL18 in Tfh cell formation in atherosclerosis.

IL18 has been shown to bind to both IL18R and Na-Cl co-transporter (NCC also known as SLC12A3).^16^ Therefore, we reconstituted lethally irradiated *Ldlr^−/−^; Rag2^−/−^* mice with 80% *CD4^Cre/+^; Bcl6^flox/flox^* + 20% *Il18r^−/−^; NCC^−/−^* (Tfh Il18 sign Ø) or 20% *Il18r^+/+^; NCC^+/+^* (WT group). Lack of IL18 signalling in Tfh led to >50% reduction of Tfh numbers (*Figure 1B*) and was associated with a significant increase in the development of atherosclerotic plaques in the aortic roots (*Figure 1C* and *D*) and aortic arches (see Supplementary material online, *Figure S5A*). There were no significant changes in serum lipid levels in both groups (see Supplementary material online, *Figure S5B–D*). Similar to the No Tfh group, abrogation of IL18 signalling in Tfh was associated with a decrease in GC B cells, IgG MDA-LDL, IgM-CuOx-LDL, IgM-MDA-LDL, and IgM T15/E06 id+ antibodies (*Figure 2A–F*), as well as an accumulation of MZB cells (*Figure 3A*), further supporting a role for Tfh–MZB cell interaction in regulating the humoral response during HF/HC diet–induced atherosclerosis.

## 4. Discussion

Tfh play essential roles in the differentiation of antigen-specific memory B cells and antibody-producing plasma cells, and dysregulation of Tfh is involved in several disease settings, including response to infectious agents, cancer, and autoimmunity,^31,32^ but little is known about the potential role of Tfh in atherosclerosis.

Clement *et al.* ^8^ showed that lack of Qa-1–restricted CD8^+^ Treg cells in *Apoe^−/−^* mice led to the accumulation of Tfh cells and acceleration of atherosclerosis, which was prevented by administration of anti-ICOSL antibody. Similarly, anti-ICOSL treatment prevented the increased accumulation of Tfh cells in MZB cell–deficient *Ldlr^−/−^* mice and the acceleration of atherosclerosis.^9^ While Clement *et al.* ^8^ did not perform any characterization of the accumulated Tfh in their model, we showed that the accumulated Tfh cells in MZB-deficient mice were poorly differentiated.^9^ Thus, it was still unclear whether normal Tfh cell development in hyperlipidaemic mice was critical for atherosclerosis development. Anti-ICOSL administration did not alter atherosclerosis development in *LDLr^−/−^* and *ApoE^−/−^* probably due to effects on other immune cells expressing ICOS. Addressing the role of Tfh cells in atherosclerosis required the use of a more selective genetic model, and this was done by Gaddis *et al*.^7^ who used a model of genetic deficiency of Tfh. They reported a slight but significant decrease in atherosclerosis in mice with No Tfh compared to controls. The apparent discrepancy between their data and ours could in part be related to the use of littermate controls in our case to control for Cre toxicity, compared to the use of C57Bl6 mice that were not littermates of *CD4^Cre/+^; Bcl6^flox/flox^* mice in their case.

In our mouse model, we have depleted all Tfh cells. Emerging evidence suggests that there are different Tfh subsets (Tfh1, Tfh2, and Tfh17). These subsets appear to have different differentiation pathways^33^ and functions.^34^ In the future, it will be interesting to develop new mouse models to interrogate the specific roles of each of these subsets in atherosclerosis.

What could be the mechanism of early atherosclerosis acceleration in the absence of Tfh cells? This cannot be explained by the profound reduction of GC B cells given that complete genetic deficiency of GC B cells was shown to be atheroprotective.^5^ Moreover, while genetic deficiency of GC B cells substantially reduces IgG antibodies, it does not affect the production of (anti-OSE) IgM antibodies.^5^ Martos-Folgado *et al.* ^6^ using a mouse with specific deletion of GC-derived plasma cells demonstrated that a substantial proportion of these IgM atheroprotective antibodies come from extrafollicular B cells. Therefore, the dramatic reduction of both IgG and IgM anti-OSE antibodies in mice with genetic Tfh deficiency strongly suggests a role for Tfh-dependent extrafollicular antibody responses in atherosclerosis, in a similar manner as in experimental immunization models using *CD4^Cre/+^; Bcl6^flox/flox^* mice have demonstrated that Tfh cells that do not enter the GC significantly contribute to extrafollicular responses.^4^ MZB cells are the prototypical B cells that engage in extrafollicular responses and are the major producers of IgM antibodies.^27,35,36^ Our data using MZB cell–deficient mice indicate that a substantial proportion of atheroprotective anti-OSE IgM^21,22,37,38^ arise from MZB cells during atherosclerosis, pointing to a determinant role of (pre-)Tfh–MZB cell interactions in this process. Indeed, we show that deletion of Tfh impairs MZB cell properties, leading to the accumulation of MZB cells with substantially altered function and antibody-secreting machinery.

Our work also identifies a previously unexplored role of MZB cells in the production of natural OSE-IgM antibodies such as the anti-PC E06/T15 antibody, which plays an important atheroprotective role.^39^ Its production has so far been associated only with B1 cells,^40,41^ but the contribution of MZB cells was overlooked. Here, we unequivocally demonstrate the important role of MZB cells in the production of anti-PC E06/T15 IgM antibody during atherosclerosis in mice. Moreover, a substantial level of this antibody production is Tfh dependent.

In our moue model, lack of Tfh leads to accumulation of ‘aberrant’ MZB cells unable to secrete anti-OSE IgM antibodies. This phenotype could result from the absence of Tfh-derived IL21, which is required for optimal antibody production by extrafollicular B cells^4^ or from co-stimulatory/inhibitory signalling pathways (i.e.PD1-PDL1). Further studies are needed to elucidate the exact mechanisms orchestrating this MZB–Tfh interaction that could be targeted to modulate the antibody secretion capability of MZB cells during atherosclerosis.

Currently, the hunt to target the inflammatory response in atherosclerosis has accelerated after the positive results of the CANTOS trial, which tested the use of canakinumab (neutralizing anti-IL1β) to treat high-risk atherosclerotic patients with prior MI. A consecutive study found that the residual inflammatory risk in patients with high cardiovascular risk and treated with canakinumab was positively correlated with IL18 levels and suggested that anti-IL18 inhibitors should be considered as potential future anti-inflammatory drugs to treat atherothrombosis.^12^ Our group was the first to show that IL18 expression was associated with human plaque instability and that IL18 inhibition was effective in reducing experimental atherosclerosis.^11^ However, our present experiments suggest that the role of IL18 in Tfh cells may limit the extent of atheroprotection associated with systemic IL18 inhibition.

Our current data show significant correlation between circulating human MZ-like B cells and levels of anti-OSE IgM antibodies, supporting a potential role for human MZB cells in the production of atheroprotective anti-OSE IgM antibodies. Human and mouse Tfh share many characteristics^3^ but circulating Tfh-like cells (cTfh) have only been described in humans and they are lacking in mouse. The role and significance of cTfh still need to be better defined.^42,43^ Despite initial reports that cTfh may resemble GC Tfh of secondary lymphoid organs,^3^ the vast majority of the current data suggest that cTfh are those that have never entered the GC and, thus, may more closely resemble pre-Tfh. The clinical cardiovascular relevance of Tfh–MZB cell interaction merits further investigation.

In conclusion, this work reveals new roles for Tfh and MZB cells regulating HF/HC-induced atherosclerosis. Tfh cell deficiency accelerates atherosclerosis, and this is associated with an altered differentiation phenotype and anti-OSE IgM antibody-producing capacity of MZB cells. We also uncover the important role of MZB cells in the production of the atheroprotective anti-OSE IgM antibodies opening a new quest to find ways we could modulate these cells to promote IgM secretion. Our work fills an important gap in our understanding of the role of these immune cell subsets in atherosclerosis and opens new lines of investigation to target Tfh–MZB cell interactions that will have important therapeutic consequences.

Our work also finds an important role of IL18 in Tfh differentiation, suggesting that the use of systemic strategies to block IL18 may increase the risk of fatal infection due to the risk of reduced Tfh cells and antibody production.

## Supplementary material

Supplementary material is available at *Cardiovascular Research* online.

## Supplementary Material

cvae027_Supplementary_Data

## Data Availability

Most of the data underlying this work are included in the manuscript and Supplementary material. The rest of the data will be shared by the corresponding author upon reasonable request.

## Notes

Translational perspective We have shown that natural IgM antibodies protect from atherosclerosis, and they are produced by MZB cells in a Tfh-dependent manner. IL18 is necessary for Tfh differentiation and consequently in the production of IgG and IgM antibodies. Thus, clinical trials targeting B cells and IL18 would have to take into consideration its role on the Tfh–MZB cell interaction.
