# Supplementary material for: Marginal zone B cells produce ‘natural’ atheroprotective IgM antibodies in a T cell–dependent manner
Source: Cardiovasc Res. 2024 Feb 21;120(3):318–28. doi: 10.1093/cvr/cvae027 (PMC10939463; doi:10.1093/cvr/cvae027)
Supplement: cvae027_Supplementary_Data [file cvae027_supplementary_data.zip › Supplementary_material_final_cleaned.docx]

**Marginal Zone B cells produce ‘natural’ atheroprotective IgM antibodies in a T cell dependent manner**

James Harrison^1^, Steve Newland, PhD^1^, Wei Jiang, PhD^1^, Despina Giakomidi, PhD^1^, Xiaohui Zhao, PhD^1^, Marc Clement, PhD^1,2^, Leanne Masters^1^, Andrej Corovic, MD^1^, Xian Zhang, PhD^3^, Fabrizio Drago, PhD^4^, Marcella Ma, PhD^5^, Maria Ozsvar Kozma^6^, Froher Yasin^1^, Yuta Saady^1^, Hema Kothari, PhD^4^, Tian X Zhao, MD, PhD^1^, Guo-Ping Shi, PhD^3^, Coleen A McNamara, MD, PhD^4^, Christoph Binder, PhD^6^, Andrew P Sage, PhD^1^, Jason M Tarkin, MD, PhD^1^, Ziad Mallat, MD, PhD^1,7^, Meritxell Nus, PhD^1*^

^1^Heart and Lung Research Institute (HLRI), Cardiovascular Division, Dept. of Medicine, University of Cambridge, United Kingdom

^2^ Laboratory for Vascular Translational Sciences (LVTS) Université de Paris, INSERM U1148, Paris, France

^3^ Department of Medicine, Brigham and Woman’s Hospital, Harvard Medical School, Boston, MA, US

^4^ Division of Cardiovascular Medicine, Department of Medicine, University of Virginia, Charlottesville, Virginia

^5^ Wellcome-MRC Institute of Metabolic Science and Medical Research Council Metabolic Diseases Unit, University of Cambridge, United Kingdom

^6^ Department of Laboratory Medicine, Medical University of Vienna, Vienna, Austria

^7^Universite de Paris, PARCC Inserm U970, Paris, France

**Supplementary Methods**

**RNAseq bioinformatics analysis :** We generated RNA-Seq datasets from sorted MZB from males and females LDLr^-/-^; Rag2^-/-^ (mice that do not have T and B cells) transplanted with donor bone marrow cells from 100% CD4^Cre/+^; Bcl6^flox/flox^ (No Tfh) or a mixed chimaera containing 80% CD4^Cre/+^; Bcl6^flox/flox^ + 20% CD4^+/+^; Bcl6^flox/flox^ or 20% *Il18r^+/+^; NCC^+/+^* (WT) (6 replicates for No Tfh and 5 replicates for WT groups, paired-end 50bp). Raw fastq data are submitted to Array Express with accession number E-MTAB-11677

(https://www.ebi.ac.uk/arrayexpress/experiments/E-MTAB-11677/). The alignment and QC were processed using the nextflow (version 21.05.0 edge^1^) pipeline nf-core/rnaseq (version 3.2, <https://nf-co.re/rnaseq>;^2^) with the option “--aligner star_salmon” and Ensembl reference genome and annotation for mouse GRCm39. For access by the editors and the reviewers to the raw RNAseq data please use this link: [https://www.ebi.ac.uk/biostudies/arrayexpress/studies/E-MTAB-11677?key=cb516519-e057-46d4-8a92-585bb386243c](https://eur03.safelinks.protection.outlook.com/?url=https%3A%2F%2Fwww.ebi.ac.uk%2Fbiostudies%2Farrayexpress%2Fstudies%2FE-MTAB-11677%3Fkey%3Dcb516519-e057-46d4-8a92-585bb386243c&data=05%7C01%7Cmn421%40cam.ac.uk%7Cd8b359e2babc47ce812c08dbe1ee3d7b%7C49a50445bdfa4b79ade3547b4f3986e9%7C1%7C0%7C638352185561812893%7CUnknown%7CTWFpbGZsb3d8eyJWIjoiMC4wLjAwMDAiLCJQIjoiV2luMzIiLCJBTiI6Ik1haWwiLCJXVCI6Mn0%3D%7C3000%7C%7C%7C&sdata=D533uT1Y8iLnfoacHsRLJOW9VBRF7MASG%2F7I%2F%2BXM3Lo%3D&reserved=0). All data will be freely available publicly once the manuscript is accepted from <https://github.com/CAD-BFX/Meri_Ziad>.

There are 55359 genes identified after next flow pipeline in total with Ensembl Gene ID annotation. These genes as input to perform the differential gene expression analysis using DESeq2 package (version 1.34.0^3^) in R (4.1.2 (2021-11-01)^4^). The design formula for DESeq2 analysis is ~Sex+Condition. After the DESeq2 analysis, 37,349 genes are removed because the missing p-adjusted values. The total number of genes for the rest of the analysis is down to 18,010. Further analysis to show the selected DEGs is presented by Heatmaps. Heatmaps are generated using R package ComplexHeatmap (version 2.10.0^5^). Fully DEGs and selected DEGs lists are in Supplementary Data 1 and available in GitHub <https://github.com/CAD-BFX/Meri_Ziad>

The gene set enrichment analysis (GSEA) performed using the web interface Enrichr (<https://maayanlab.cloud/Enrichr/>) with 515 up regulated and 543 down regulated DEGs list, respectively. Selected pathways using Reactome 2022, Kegg 2021 and Panther 2016 are plotted in Figure 3E and all pathways can be found in Supplementary Data 2.

**Supplementary Table 1**. Mouse flow cytometry and human mass cytometry antibodies

| Target | Clone | Company |
| --- | --- | --- |
| B220 | RA3-6B2 | eBioscience |
| IgM | II-41 | eBio |
| CD23 | B3/B4 | eBioscience |
| CD21 | eBio4E3 | eBioscience |
| CD44 | 1M7 | Biolegend |
| CD4 | RM4-5 | Biolegend |
| CD3 | 145-2C11 | Biolegend |
| CD62L | MEL-14 | Biolegend |
| CD95 | Jo2 | BD |
| GL7 | GL7 | eBioscience |
| B220 | RA3-6B2 | eBioscience |
| CD19 | 1D3 | BD |
| CD138 | 281-2 | Biolegend |
| CD4 | RM4-5 | Biolegend |
| CXCR5 | L138D7 | Biolegend |
| ICOS | C398.4A | eBioscience |
| PD1  CD274 | RMP1-30  MIH5 | Biolegend  eBioscience |
| CD11b | M1/70 | Biolegend |
| CD11c | N418 | Biolegend |
| CD25 | PC61 | Biolegend |
| CD5 | 53-7.3 | BD |
| F4/80 | BM8 | Thermo Fisher |
| Foxp3 | FJK16s | Thermo Fisher |
| Ly6B2 | HK1.4 | Biolegend |
| Ly6G | RB6-8C5 | Thermo Fisher |
| hIgD | IA6-2 | Fluidigm |
| hCD27 | O321 | Fluidigm |
| hCD19 | HIB19 | Fludigm |
| hCD20 | 2H7 | Fluidigm |
|  |  |  |

**Supplementary Table 2**:

| **Cell population** | **Antibody markers** |
| --- | --- |
| Germinal centre (GC) | B220^+^ Gl7^hi^ CD95^hi^ |
| Plasma cells | B220^+^ CD138^+^ CD43^+^ |
| Marginal Zone B cells (MZB) | B220^+^ IgM^+^ CD21^+^ CD23^lo^ |
| Follicular B cells | B220^+^ IgM^+^ CD21^lo^ CD23^+^ |
| B1a cells | B220^+^ CD5^+^ CD11b^+^ |
| B1b cells | B220^+^ CD5^-^ CD11b^+^ |
| T effectors (TEM) | CD3^+^ CD4^+^ CD44^+^ CD62L^-^ |
| T follicular helper cells (Tfh) | CD3^+^ CD4^+^ CD44^+^ CD62L^-^ CXCR5+ PD1^+^ ICOS^+^ |
| T follicular regulatory cells (Tfr) | CD3^+^ CD4^+^ CD44^+^ CD25^+^ CXCR5^+^ PD1^+^ Foxp3^+^ |
| T regulatory cells (Tregs) | CD3^+^ CD4^+^ CD44^+^ CD25^+^ CXCR5^-^ Foxp3^+^ |
| CD4 Th1 cells | CD4^+^ IFNg^+^ |
| CD4 Th17 cells | CD4^+^ IL17^+^ |
| Neutrophils | CD11c^-^ NK.K1^-^ Ly6G^+^ |
| Macrophages | CD11c^-^ NK.K1^-^ Ly6G^-^ Ly6C^+^ F4/80^+^ |
| Monocytes | CD11c^-^ NK.K1^-^ Ly6G^-^ Ly6C^hi/lo^ F4/80^-^ CD11b^+^ |
| Eosinophils | CD11c^-^ NK.K1^-^ Ly6G^-^ Ly6C^-^ |
| Human unswitched B cells | CD45^+^ CD66b^-^ CD56^-^ CD14^-^ CD3^-^ CD19^+^ CD20^+^ IgD^+^ CD27^+^ |

**Supplementary Table 3**: Primers used for qRT-PCR

|  | Sense | Antisense |
| --- | --- | --- |
| *Cd274* | CTTCCTTCCTTCCTTCCT | GCATAGTGAGCAACCATT |
| *36B4* | TCTCCAGTGGCTCCATTGA | CTCGCTGGCTCCCACCTT |
| *Gapdh* | CCCACTCCTCCACCTTTGAC | CCACCACCCTGTTGGTGTA |
| *Pdl2* | CCGGCCTGCACCATCGCTTT | TCCCAAGACCGCAGGTCCAGAT |
| *April* | CCTCACTTCTGAGACCACAGC | GAACAACAGTCAAGGCAAAGC |
| *Baff* | CAGGAACAGACGCGCTTTC | GTTGAGAATGGCGGCATCC |
| *Il5* | CAAGCAATGAGACGATGAGGC | CCCACGGACAGTTTGATTCTTC |
| *Tlr6* | TGGATGTCTCACACAATCGG | GCAGCTTAGATGCAAGTGAGC |
| *Atf3* | GGAATATGGAATGAGAACA | CATCTAAATAGCACAACAC |
| *Mdm2* | CCGAGTTTCTCTGTGAAGGAGC | GTCTGCTCTCACTCAGCGATGT |
| *Map2k7* | TCAGGTGTGGAAGATGCGGTTC | AAGGGCAGTCATGGCTCTTGAG |

**Supplementary Table 4**. Baseline clinical characteristics and study criteria for human blood samples

| **Study name** | Residual Inflammation and Plaque Progression Long-Term Evaluation (RIPPLE) study | Rituximab in Patients With Acute ST-elevation Myocardial Infarction Study | The Coronary Assessment in Virginia cohort (CAVA) study |
| --- | --- | --- | --- |
| **Ethical approval** | 19/EE/0043 | 16/EE/0241 | HSR #15328 |
| **Trial registration number** | NCT04073810 | NCT03072199 |  |
| **n** | 16 | 21 | 20 |
| **Summary of inclusion criteria** | Men and women >18 years with recent myocardial infarction within 2 weeks. | Men and women aged 18 to 75 years with ST-segment elevation myocardial infarction and successful primary percutaneous coronary intervention during the first 24 h of cardiac chest pain onset | Men and women with stable coronary artery disease undergoing coronary angiography |
| **Age, mean (SD)** | 61.5 (7.41) | 58.9 (10.1) | 66.85 (9.52) |
| **Sex male, n (%)** | 11 (69%) | 19 (90%) | 11 (55%) |
| **Hypertension, n (%)** | 7 (44%) | 9 (43%) | 12 (60%) |
| **Hypercholesterolaemia, n (%)** | 7 (44%) | 6 (29%) | 13 (65%) |
| **Diabetes mellitus, n (%)** | 1 (6%) | 2 (10%) | 6 (30%) |
| **Current smoker, n (%)** | 5 (31%) | 6 (29%) | 3 (15%) |
| **Aspirin, n (%)** | 16 (100%) | 21 (100%) | 13 (65%) |
| **Statins, n (%)** | 16 (100%) | 21 (100%) | 13 (65%) |
| **Beta-blocker, n (%)** | 15 (95%) | 17 (81%) | 10 (50%) |
| **P2Y_12_ inhibitors, n (%)** | 16 (100%) | 21 (100%) | 1 (5%) |
| **ACE inhibitors, n (%)** | 16 (100%) | 19 (90%) | 1 (5%) |
| **Diuretics, n (%)** | 1 (6%) | 0 (0%) | 6 (30%) |
| **Novel oral anticoagulants, n(%)** | 2 (13%) | 0 (0%) | 2 (10%) |
| **Proton Pump Inhibitors, n (%)** | 16 (100%) | 21 (100%) | 8 (40%) |


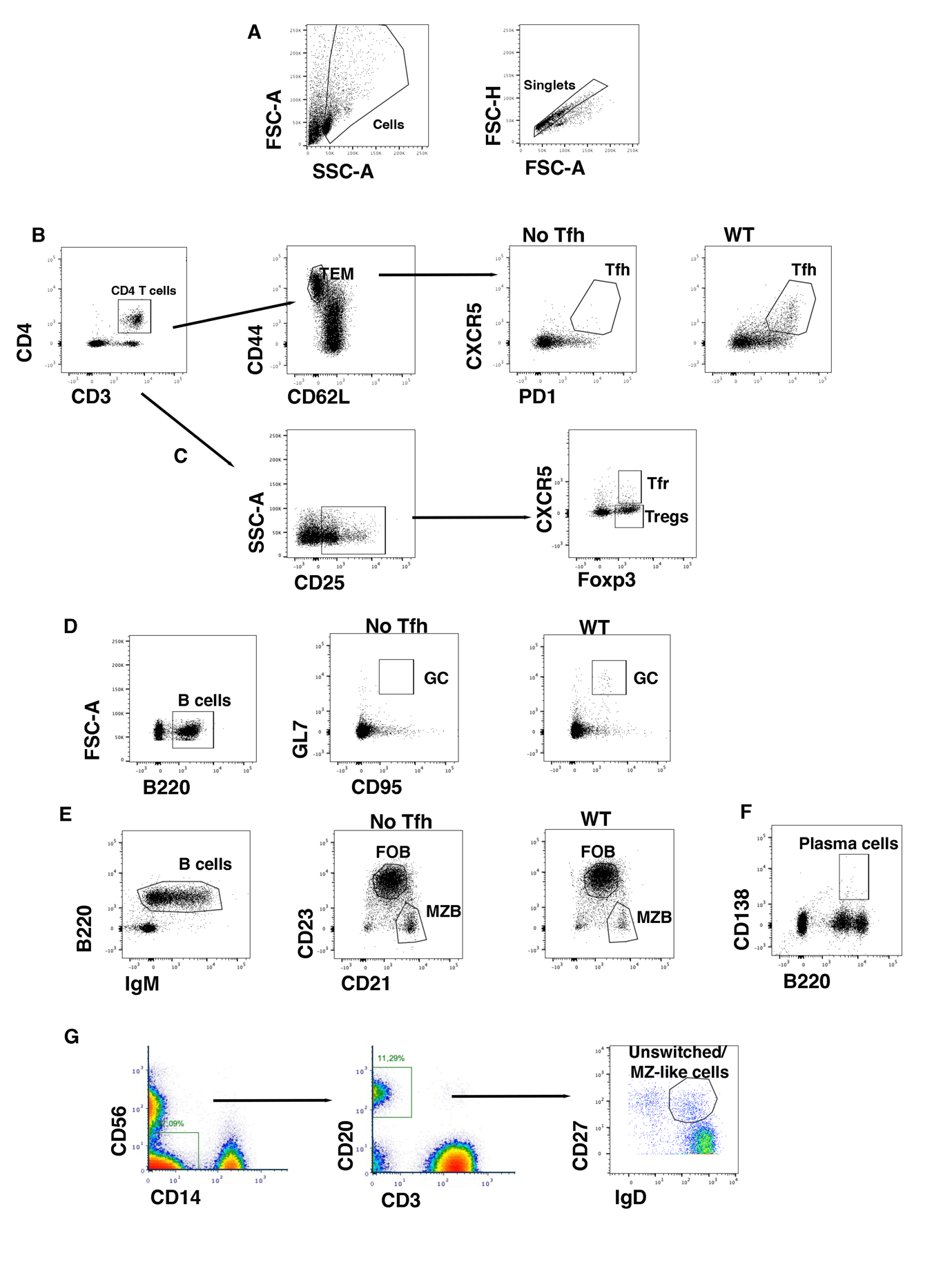
**Supplementary Figure 1. Representative flow cytometry plots for different immune cell subsets.** Representative plots of gating strategies to identify different immune cell subsets in mouse (A-E) and humans (F). In some cases a representative plot for a WT and No Tfh mouse are also shown (A, C-D): (A) Singlets; (B) spleen TEM and Tfh cells; (C) spleen Tregs and Tfr cells; (D) spleen GC B cells; (E) spleen MZB cells; (F) bone marrow plasma cells; (G) human blood unswitched B cells. Human B cells gating was performed following the Technical Note from Fluidigm available at https://www.imc.unibe.ch/unibe/portal/fak_medizin/micro_imc/content/e987276/e1010628/ApproachtoBivariateAnalysis_TechnicalNote400248B1.pdf. Look at Supplementary Table 2.


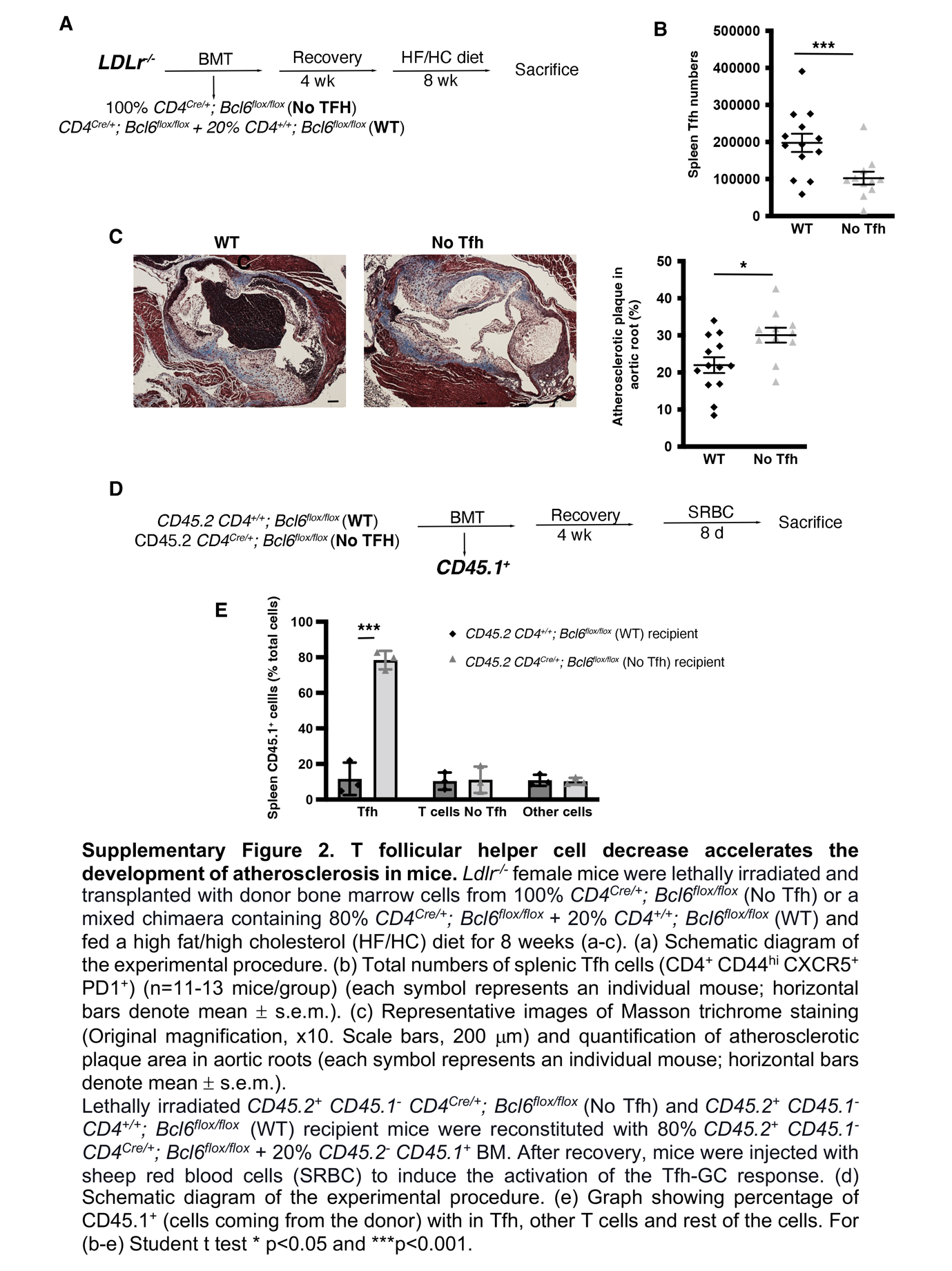


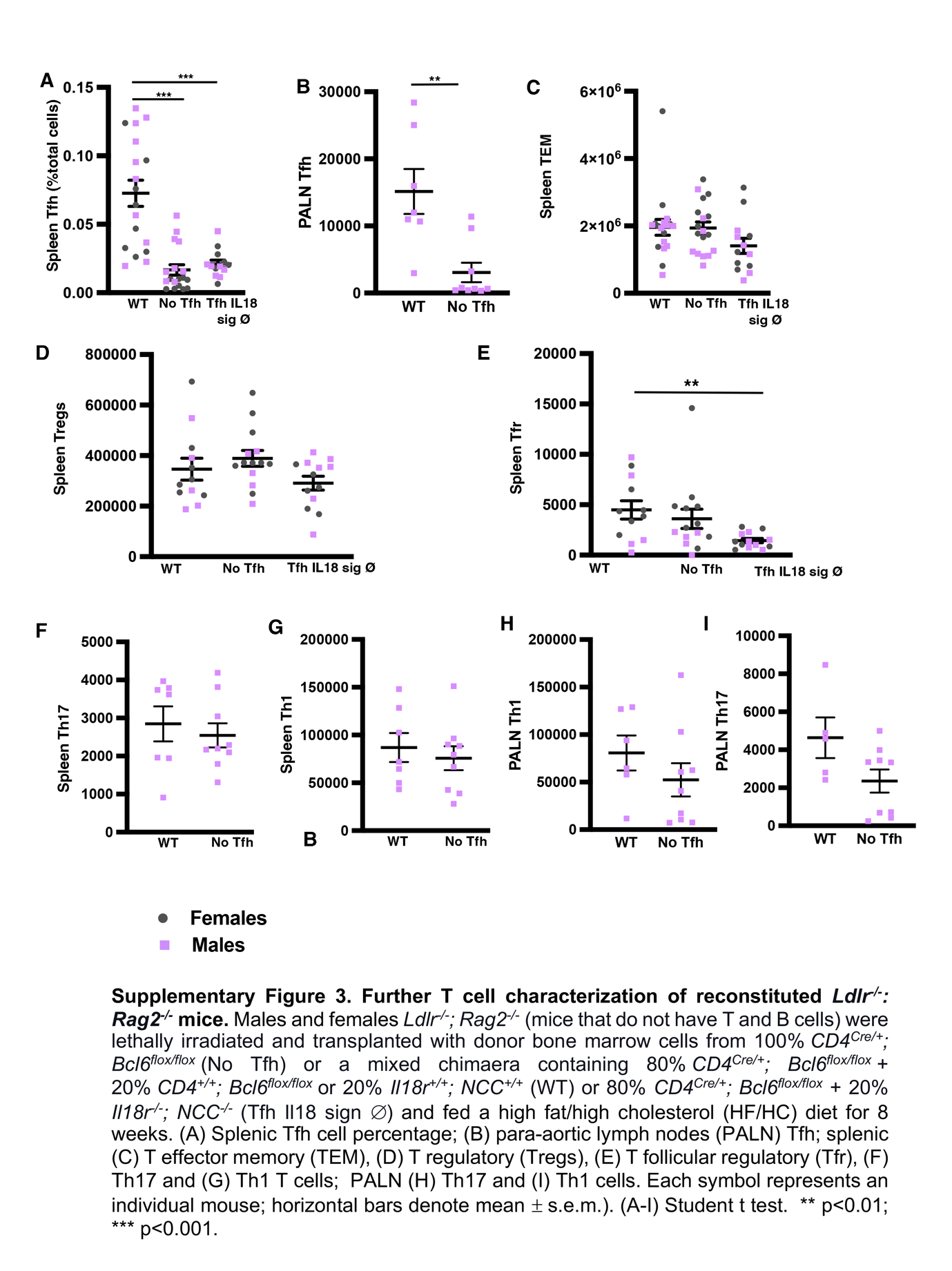


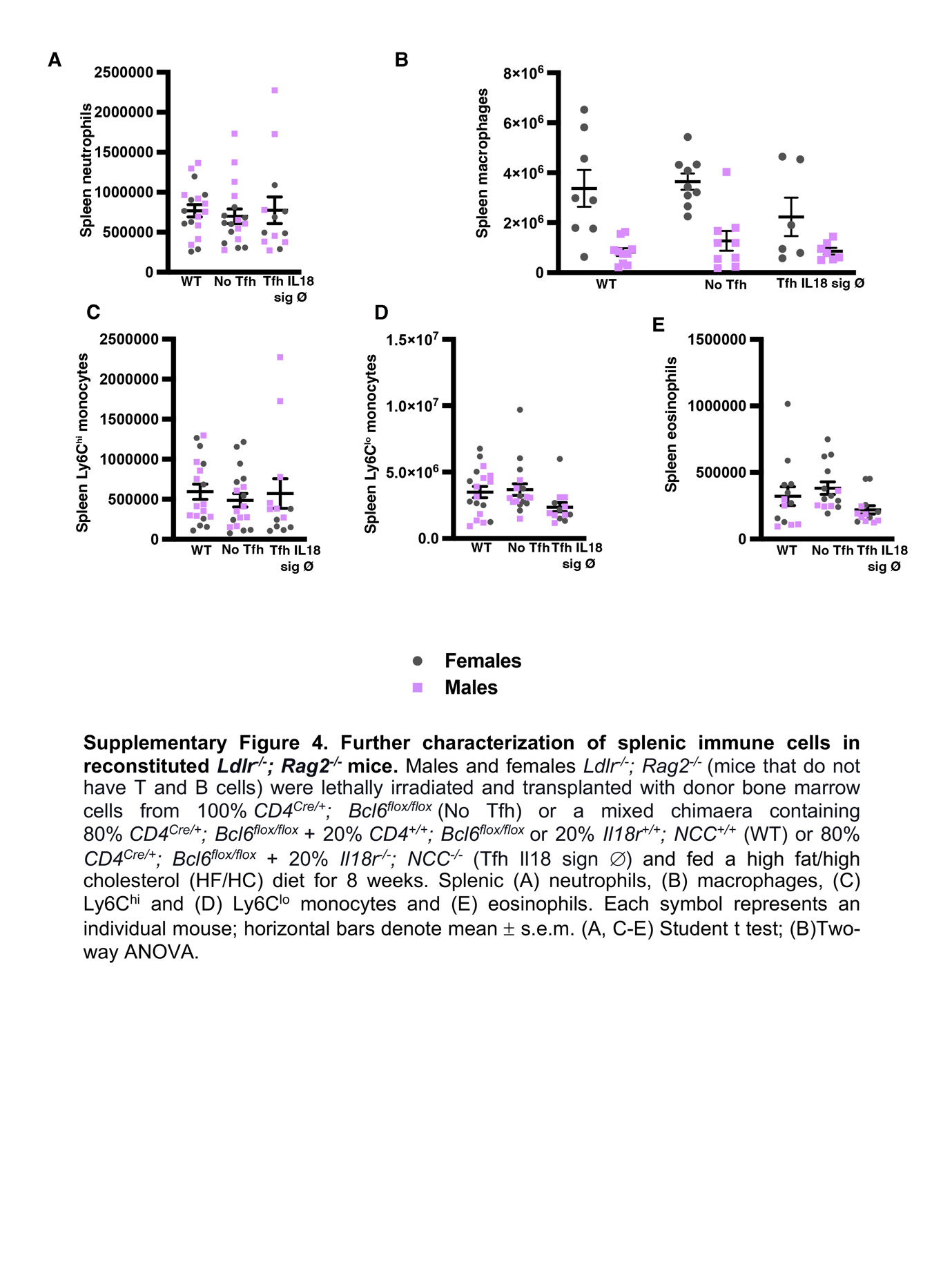


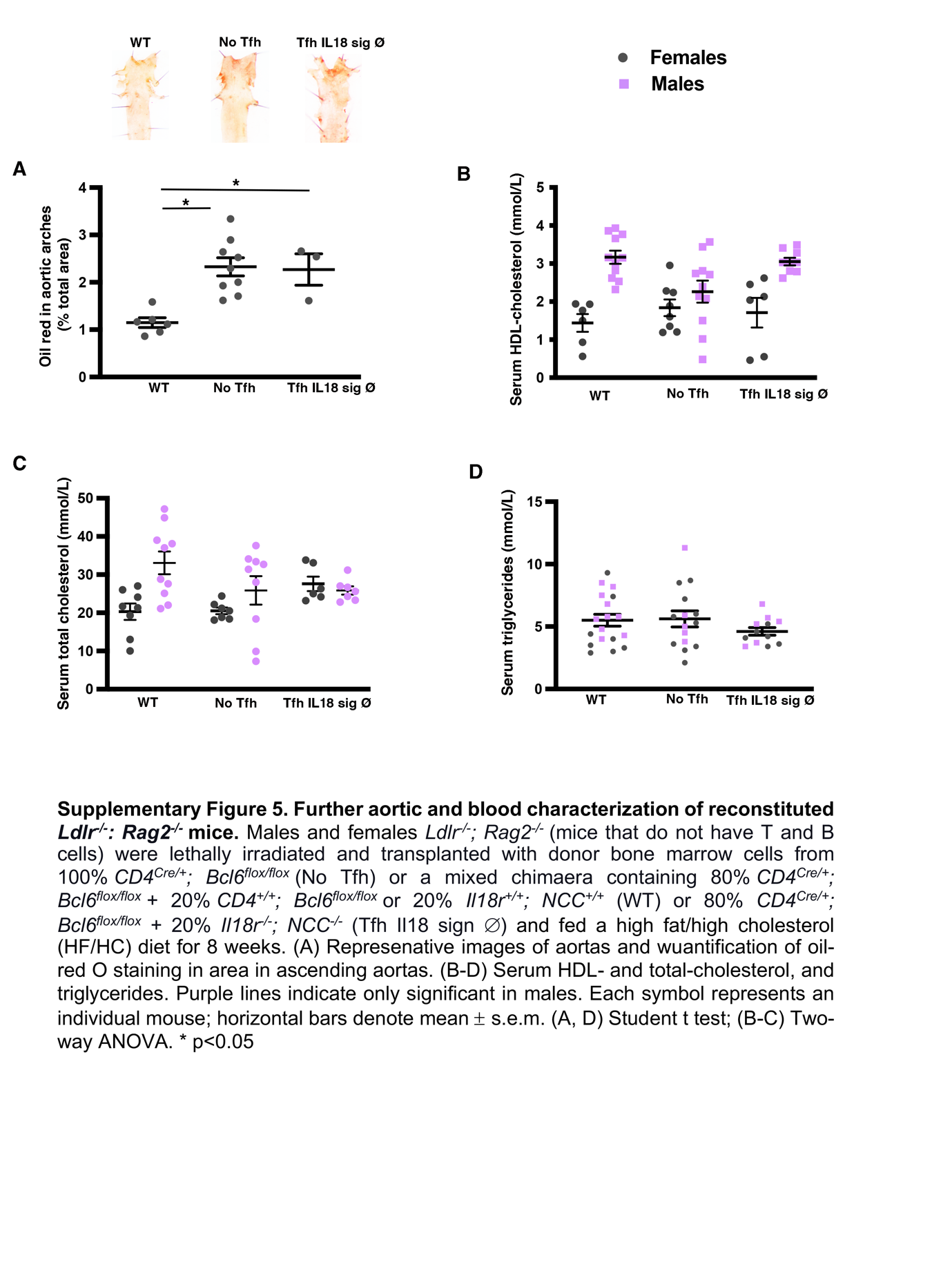


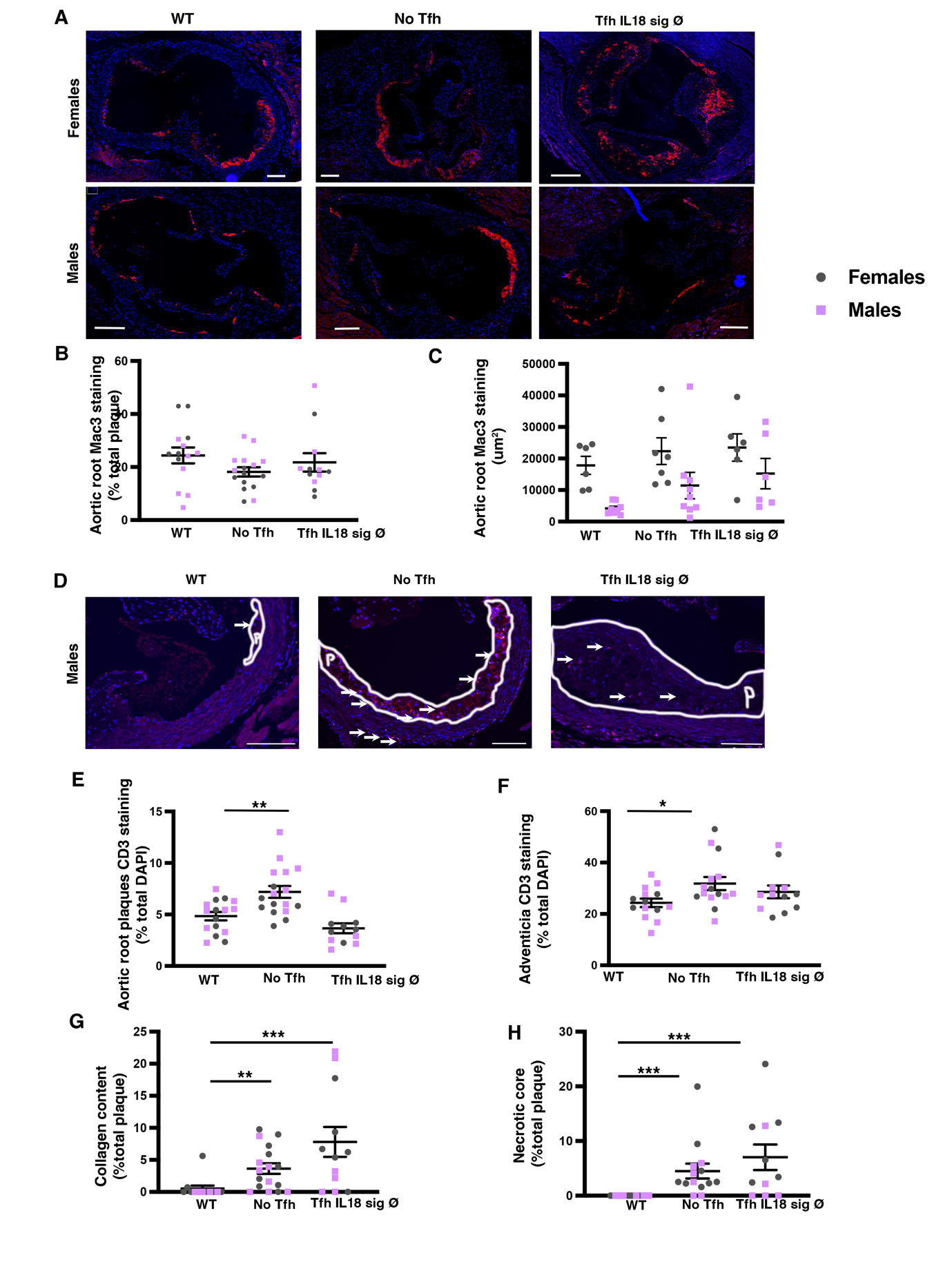


**Supplementary Figure 6. Further atherosclerotic plaque characterization of reconstituted** *Ldlr^-/-^; Rag2^-/-^* **mice.** Males and females *Ldlr^-/-^; Rag2^-/-^* (mice that do not have T and B cells) were lethally irradiated and transplanted with donor bone marrow cells from 100% *CD4^Cre/+^; Bcl6^flox/flox^* (No Tfh) or a mixed chimaera containing 80% *CD4^Cre/+^; Bcl6^flox/flox^* + 20% *CD4^+/+^; Bcl6^flox/flox^* or 20% *Il18r^+/+^; NCC^+/+^* (WT) or 80% *CD4^Cre/+^; Bcl6^flox/flox^* + 20% *Il18r^-/-^; NCC^-/-^* (Tfh Il18 sign ∅) and fed a high fat/high cholesterol (HF/HC) diet for 8 weeks. Aortic root sections were stained with Mac3 to detect macrophages (A-C) and CD3 to detect T cells (D-F). Representative Mac3 (A) and CD3 (D) micrographs of atherosclerotic plaques. Relative (B) and total (C) plaque area positive for Mac3. Relative plaque area positive for CD3 (E, F). (G) Collagen (from Masson Trichrome blue staining) positive staining and (H) quantification of necrotic cores in atherosclerotic plaques of aortic roots. Scale bars: 100 uM. Each symbol represents an individual mouse and 2 representative sections from each mouse were analyzed; horizontal bars denote mean ± s.e.m. (B, E-H) Student t test; (C) Two-way ANOVA. * p<0.05; ** p<0.01; ***p<0.001.


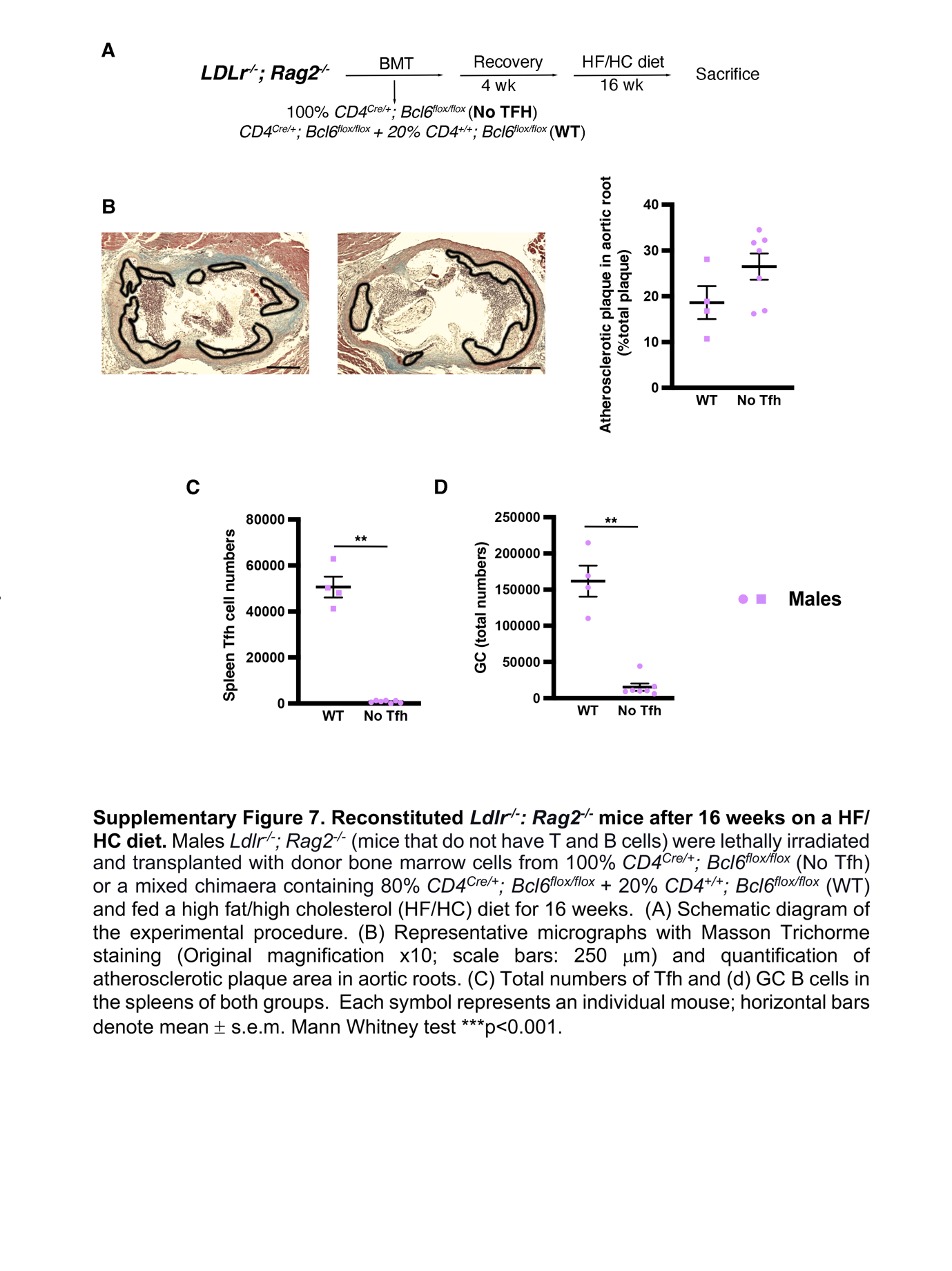


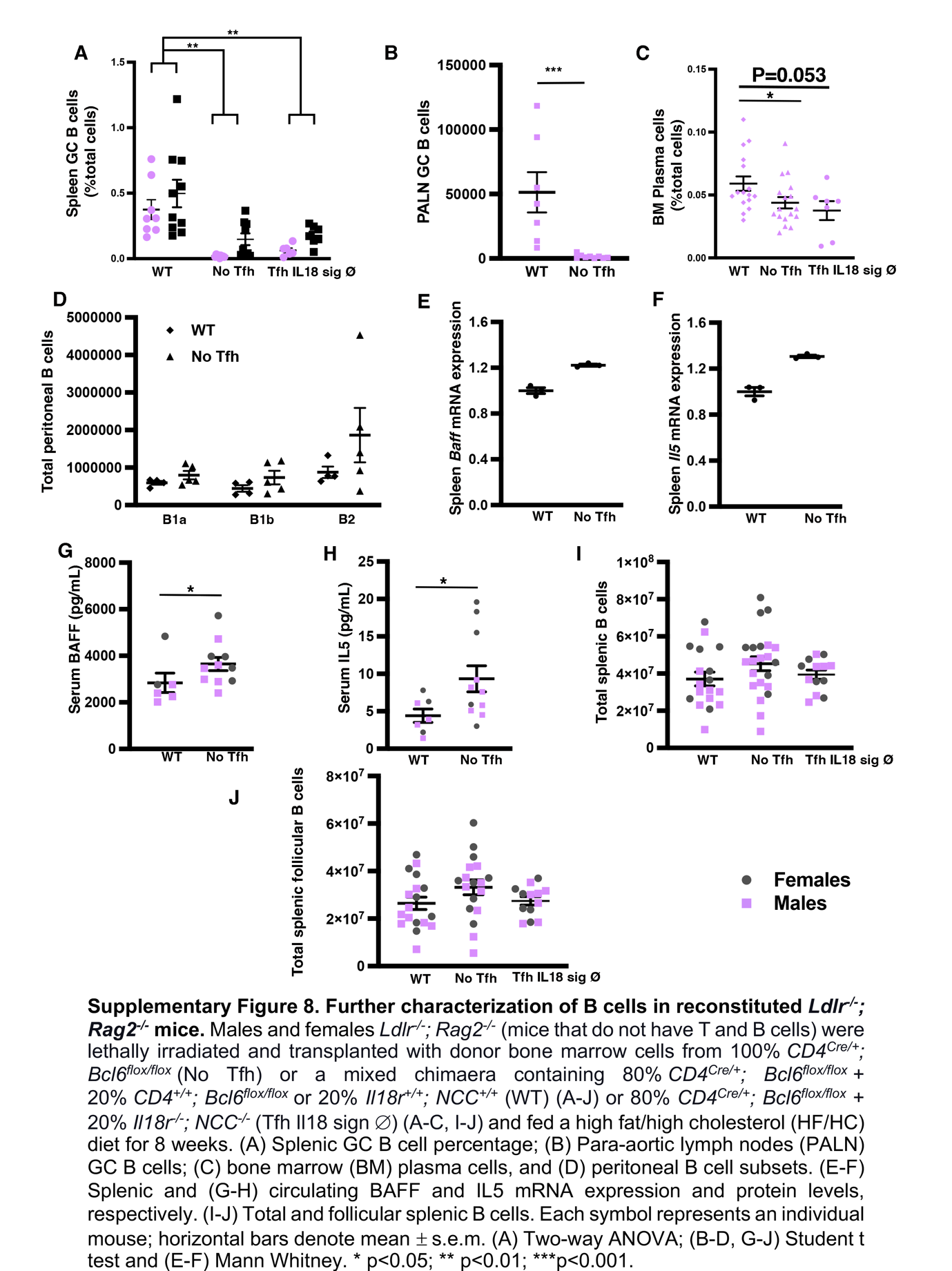


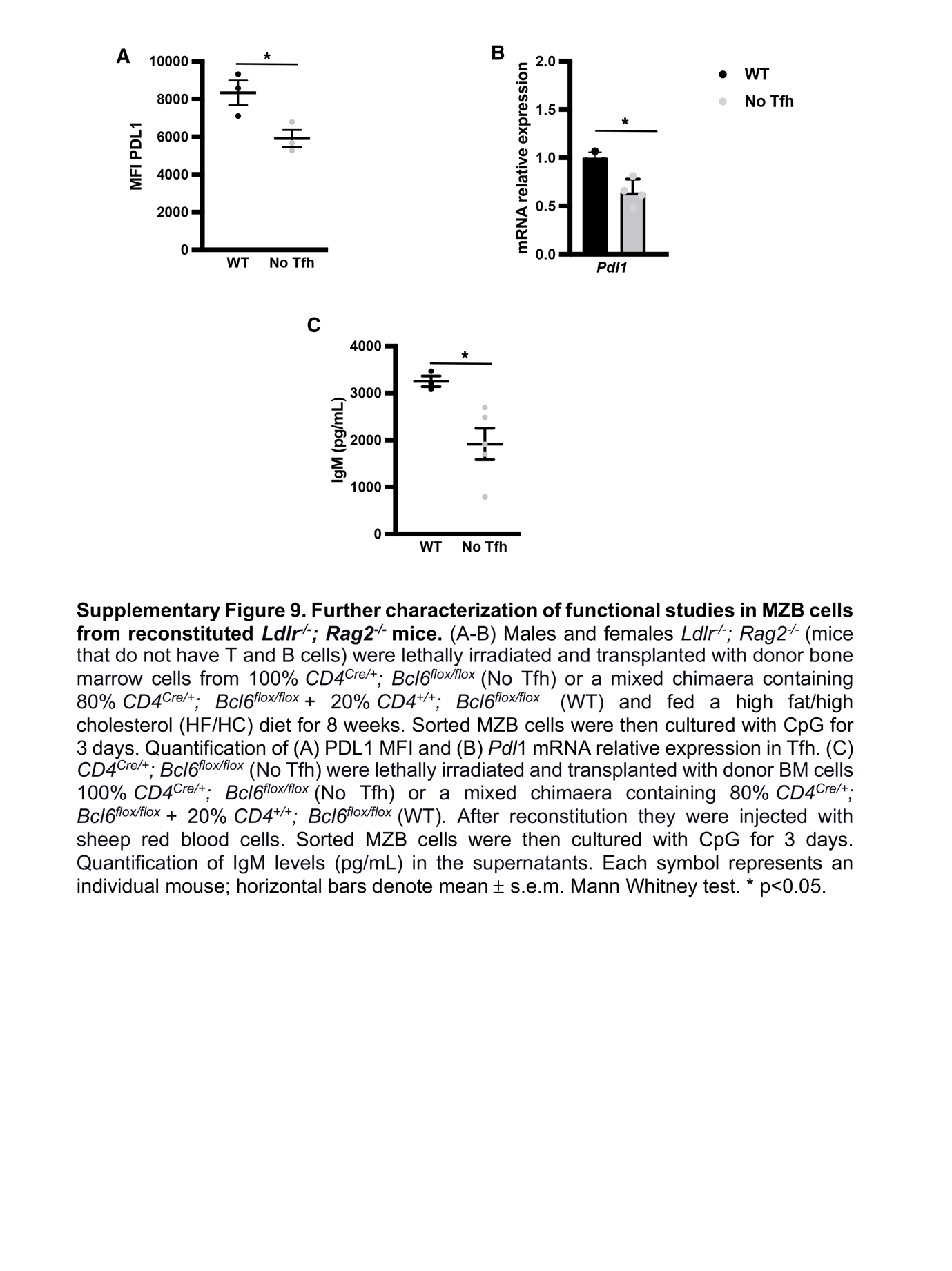


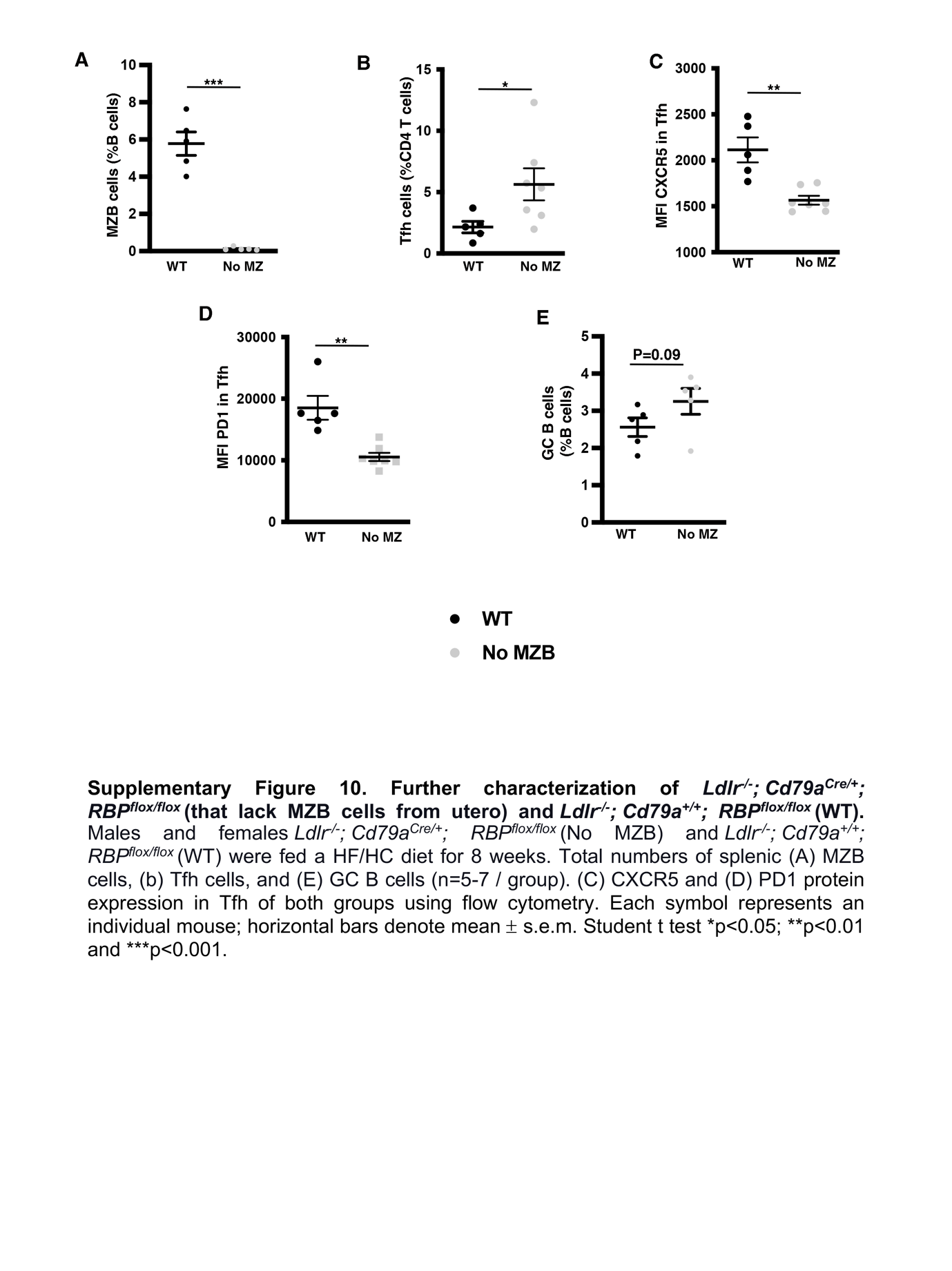


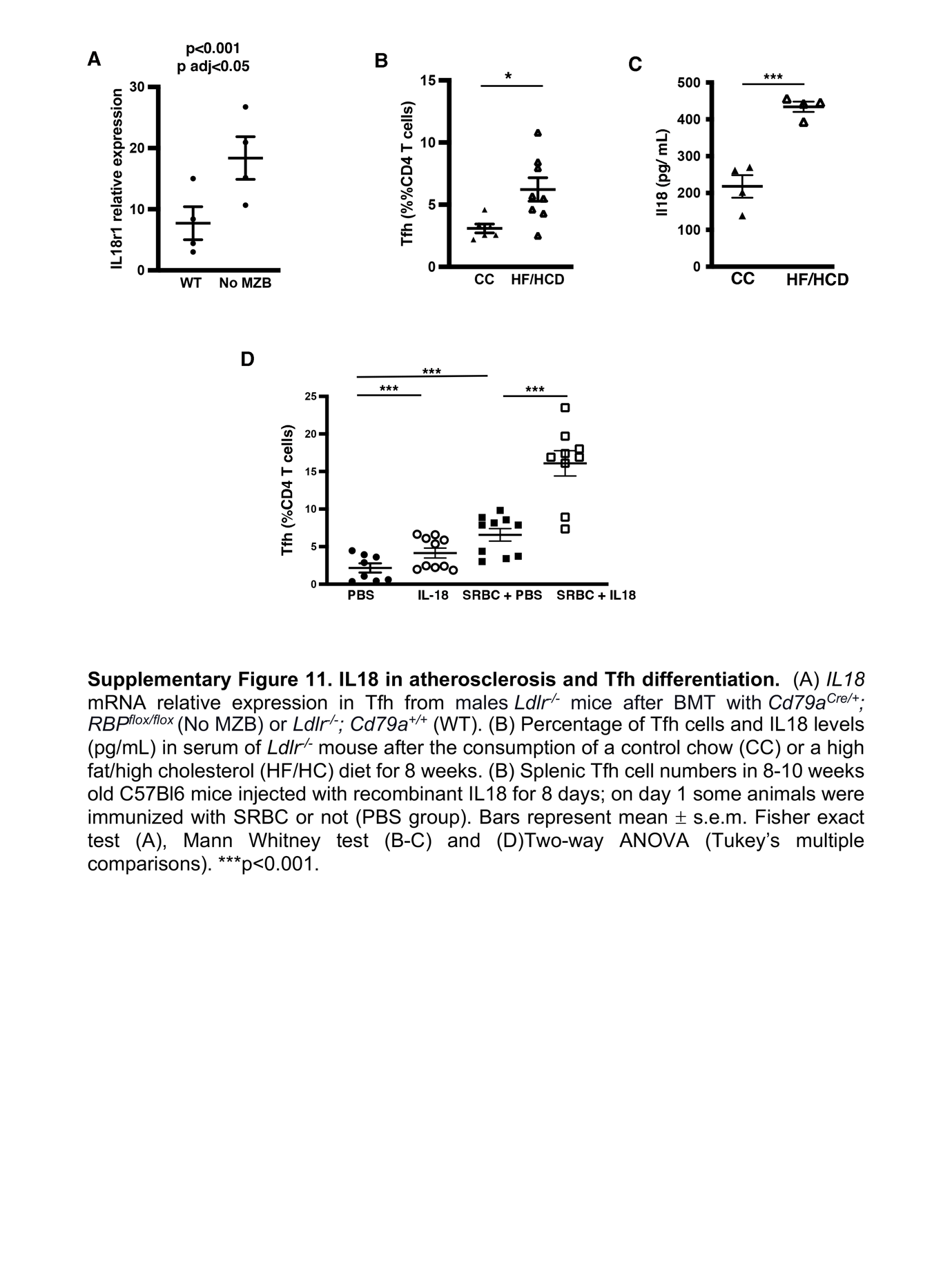


**Supplementary references**

1. Di Tommaso P, Chatzou M, Floden EW, Barja PP, Palumbo E, Notredame C. Nextflow enables reproducible computational workflows. *Nat Biotechnol*. 2017;35:316–319.

2. Ewels PA, Peltzer A, Fillinger S, et al. The nf-core framework for community-curated bioinformatics pipelines. *Nat Biotechnol*. 2020;38:276–278.

3. Love MI, Huber W, Anders S. Moderated estimation of fold change and dispersion for RNA-seq data with DESeq2. *Genome Biol*. 2014;15:550.

4. Team RC. R: A language and environment for statistical computing. R Foundation for Statistical Computing, Vienna, Austria. Available online at https://www.R-project.org/.

5. Gu Z, Eils R, Schlesner M. Complex heatmaps reveal patterns and correlations in multidimensional genomic data. *Bioinformatics*. 2016;32:2847–2849.
